# The effect of curculigo orchioides (Xianmao) on kidney energy metabolism and the related mechanism in rats based on metabolomics

**DOI:** 10.1002/fsn3.2573

**Published:** 2021-09-13

**Authors:** Limei Chen, Baohua Qu, Hui Wang, Hongning Liu, Yongmei Guan, Juanqing Zhou, Jiaqi Zhang

**Affiliations:** ^1^ The Affiliated Hospital of Jiangxi University of CM Nanchang China; ^2^ Jiangxi University of Chinese Medicine Nanchang China; ^3^ Nanchang Institute of Science & Technology Nanchang China

**Keywords:** kidney energy metabolism, metabolomics, RT‐PCR, Xianmao

## Abstract

The Chinese materia medica Xianmao (XM) is widely used in Chinese clinics and the traditional Chinese medicine diets. Although XM is often used to study its kidney‐yang effect, the research on its effect on kidney energy metabolism and its mechanism is still relatively lacking. In this study, rats were given different doses of XM water extract for 4 weeks. Biochemical method was used to detect the content of serum biochemical indexes of liver and kidney function and blood lipid indicators, and HE staining method was used to observe the histopathological of liver and kidney in rats. The kidney Na^+^‐K^+^‐ATPase, Ca^2+^‐Mg^2+^‐ATPase, SDH (succinate dehydrogenase) enzyme activity, and the content of ATP in rats were measured. Metabolomics technology was used to analyze the potential biomarkers related to the effects of XM on kidney energy metabolism, and then, the metabolic pathways were analyzed. RT‐PCR was used to detect the expression of Ampk, Sirt1, Ppar‐α, and Pgc‐1α mRNA in kidney of rats. The results showed, compared with the blank control group, there was no significant effect on liver and kidney function in XMH, XMM, and XML groups. These significantly increased the kidney Na^+^‐K^+^‐ATPase, Ca^2+^‐Mg^2+^‐ATPase, SDH enzyme activity, and ATP content in XMH, XMM, and XML groups. Mitochondrial metabolic rate was inhibited in XMH group, but it was significantly increased in XMM and XML groups. The number of mitochondria was increased in XMH, XMM, and XML groups. Overall, these effects may be mediated by TCA cycle metabolism, butanoate metabolism, propanoate metabolism, alanine, aspartate, and glutamate metabolism, retinol metabolism, purine metabolism, pentose phosphate metabolism, aminoacyl‐tRNA biosynthesis, valine, leucine, and isoleucine biosynthesis, and degradation metabolism pathways, as well as by increasing expression of upstream genes Ampk, Sirt1, Ppar‐α, and Pgc‐1α mRNA.

## Graphical abstract

In this study, we investigated the effects of XM on kidney energy metabolism and the effects of XM on kidney function in rats. These results suggested that XM could promote kidney energy metabolism and have no significant effect on the function of liver and kidney in this experimental conditions.

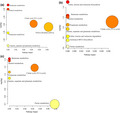

## 1 INTRODUCTION

The traditional Chinese medicine is widely used in Chinese clinics, and a variety of molecular mechanisms are associated with functional regulation mediated by Chinese materia medica (Hu et al., 2020; Wang et al., 2019). To balance the yin and yang, the traditional Chinese medicine diets or combining foods with certain Chinese materia medica are purposefully used for the patients (Zhou et al., 2021). One of the Chinese materia medica, Xianmao (root tubers of Curculigo orchioides, belonging to the family Amaryllidaceae) often utilized with foods to reverse the kidney‐yang deficiency symptoms, such as the decline of vital gate fire, frequent urination, cold extremities, sore lower back, waist and knee pain, and soft bones (Chauhan et al., 2010). In addition, XM could active the TRPV1 of rat DRG ganglion cells, which reflected the heat properties of XM (Li et al., 2018). The physiological function of the body could be affected through the flow of matter and energy. Energy metabolism was considered to be one of the basic forms of the body's material metabolism. The cold and heat properties of the drug were closely related to the body's energy metabolism (Wang et al., 2008). The main active components of XM are curculigo orchioides phenolic glycoside, among which curculigoside and tenoside are higher in content (Yang, 2012), and curculigoside has a certain protective effect on the function of mitochondria (Zhao et al., 2020). Studies have shown that XM can affect the energy metabolism of normal rats (Fan, 2010). It was found that XM could regulate the metabolism of substances in the body, cyclic nucleotides, and endocrine content (Li et al., 2012), reduce the content of TG and cGMP, and increase the content of cAMP/cGMP, T3, T4, TSH, Ts, Glu, TC, TP, etc. in rat serum (Zhou et al., 2014), to improve the symptoms of kidney‐yang deficiency model in rats. In China, although Xiaomao (XM) is often used to study its kidney‐yang effect, the research on its effect on kidney energy metabolism and its mechanism is still unknown.

Metabolomics is a technology for analyzing small molecules in organisms based on high‐throughput, multivariate data. It can fully reflect the profile and level changes of endogenous metabolites in the body. And it can amplify small differences in upstream gene and protein expression, and further in‐depth analysis of the metabolic pathways related to different metabolites can explain the mechanism of action of the research object (Wu et al., 2021). This technology has been widely used in food science (Chen et al., 2020), medicine (Wang, Zhang, et al., 2020; Wang, Chen, et al., 2020; Wang, Gong, et al., 2020; Wang, Jia, et al., 2020) and other fields. Nontargeted metabolomics can comprehensively and unbiasedly reflect the metabolic state of small molecules in organisms, which is conducive to the screening of biomarkers and the construction of dynamic metabolic networks in organisms (Wang, Zhang, et al., 2020; Wang, Chen, et al., 2020).

In this study, pharmacological experiments combined with nontargeted metabolomics technology were used to explore the effects of XM on kidney energy metabolism and its mechanism. This will improve our understanding of XM and provide a scientific basis for its use.

## 2 METHODS

### 2.1 Medicinal materials and reagents

Xianmao (Jiangzhong Chinese medicine decoction pieces company, batch number: 190401) was identified as the dry root tubers of curculigo orchioides by Jiangxi University of Chinese Medicine Appraisal Department. ATPase kit (Nanjing Jiancheng Bioengineering Institute batch number: 20191230), SDH kit (Nanjing Jiancheng Bioengineering Institute, batch number: 20191224), ATP kit (Beyotime, S0027), Uric acid kit (Nanjing Jiancheng Bioengineering Institute, batch number: 20190605), Creatinine kit (Nanjing Jiancheng Bioengineering Institute, batch number: 20190606), Urine nitrogen kit (Nanjing Jiancheng Bioengineering Institute, batch number: 20190606), Alanine transaminase kit (Nanjing Jiancheng Bioengineering Institute, batch number: 20190610), Aspartate aminotransferase kit (Nanjing Jiancheng Bioengineering Institute, batch number: 20190606), Triglyceride kit (Hunan haiyuan medical technology Co., Ltd, batch number: 20201027), Total cholesterol kit (Hunan haiyuan medical technology Co., Ltd, batch number: 20201007), Low density lipoprotein (Hunan haiyuan medical technology Co., Ltd, batch number: 20201008), High density lipoprotein (Hunan haiyuan medical technology Co., Ltd, batch number: 20201008), Total bile acid (Hunan haiyuan medical technology Co., Ltd, batch number: 20201109), RNA extract (solarbio, G3013), Extracellular oxygen consumption kit (Abcam, ab197243), NAO (Shanghai kangxiang biological technology Co., Ltd, M × 430), RIPA (Solarbio, R0020), Servicebio®RT First Strand cDNA Synthesis Kit (Servicebio, G3330), and SYBR Green qPCR Master Mix (Servicebio, G3322).

### 2.2 Instrument

Multimode reader (Tecan, Switzerland, Spark 10M), Tissue Lyser (Spex, Geno 2010), Pathological slicing machine (Leica, RM2016), Microscope (Nikon, Eclipse Ci‐L), Automatic biochemical analyzer (Beckmancoulter, AU480), UHPLC (Agilent, 1290), Q‐TOF/MS (Agilent, 6538), Centrifugal thickener (Thermo Fisher, SPD131DDA‐PI‐230), and Fluorescence quantitative PCR (Stepone plus, ABI).

### 2.3 Extraction of XM

Xiaomao (XM) was soaked in 10 times water for 60 min, refluxed for 60 min, and then poured out. The residue was extracted by 8 times water reflux for 40 min. The two filtrates were mixed and concentrated into 1 g/ml solution, which was diluted for standby before administration.

### 2.4 Experimental animals and animal processing

Thirty‐two male *SD* rats, 160 ± 20 g, obtained from Hunan Slack Jingda Experimental Animals Co., Ltd., were allowed to acclimatize for 7 days in the experimental animal science and technology center of Jiangxi University of Chinese Medicine. The temperature was set to 24 ± 2°C. The humidity was set between 55% and 65%, and the lighting condition was set to 12‐hr light–dark alternating. Rats were fed with standard diet and drink. The study was approved by the Experimental Animal Ethics Sub‐Committee of the Academic Committee of Jiangxi University of Chinese Medicine and complies with the animal research guidelines of the China Ethics Committee (JZLLSC2019‐0082).

In total, 32 rats were randomly divided into the blank control group (Control), high dose of XM group (XMH), medium dose of XM group (XMM), and low dose of XM (XML), with eight rats in each group. The XMH, XMM, and XML groups were administered the crude extracts of 3.15, 1.05, and 0.35 g/kg by gavage for 4 weeks, respectively. The control group was given the same volume of saline. After the experiment, rats were fasting for 12 hr. The feces of the rats were collected before dissection. The kidney tissue was collected and stored in a −80° refrigerator.

### 2.5 Effects of XM on ALT, AST, UA, BUN, Cr, TG, TC, TBA, HDL‐C, and LDL‐C

The serum was taken to measure the concentrations ALT (alanine aminotransferase), AST (aspartate aminotransferase), UA (uric acid), BUN (urine nitrogen), Cr (creatinine), TG (Triglyceride), TC (total cholesterol), TBA (total bile acid), HDL‐C (high density lipoprotein cholesterol), and LDL‐C (low density lipoprotein cholesterol) following the manufacturer's instructions.

### 2.6 Effect of XM on kidney and liver pathological sections

The kidney and liver tissues were dissected, conventionally taken, dehydrated, embedded, prepared, and stained with HE. Then, they were observed and described under an optical microscope. Different types of lesions in the main description were photographed.

### 2.7 Determination of Na^+^‐K^+^‐ATPase, Ca^2+^‐Mg^2+^‐ATPase, and SDH enzyme activity in rat kidney tissue

Physiological saline was added to kidney tissue to make a 10% homogenate, centrifuged for 10 min (4°C, 211 g). The supernatant was obtained to measure the enzyme activity of kidney tissue according to the manufacturer's instructions. Coomassie Brilliant Blue method was used to determine the protein concentration of tissues.

### 2.8 Determination of ATP content

0.2 g of kidney tissue sample was weighed, and 1 ml of lysate was added. After homogenizing on ice, it was left for about 5 min to fully lysate. Then, the supernatant was centrifuged for 5 min (4°C, 12,000 *g*), and the supernatant was taken for use. The ATP content of rat kidney was detected according to the manufacturer's instructions.

### 2.9 Mitochondrial oxygen consumption rate

The kidney mitochondria were extracted by differential centrifugation. The mitochondrial oxygen consumption kit was used to detect the signal value of mitochondria, and the CurveExper 1.4 software was used to draw the mitochondrial oxygen consumption curve of each rat, and the slope of the curve was calculated.

### 2.10 NAO staining to observe the number of mitochondria

10‐mercaptoacridine orange (NAO) is a reagent that can specifically bind to mitochondria. It can be used to detect the specific fluorescent markers of mitochondria and was often used to detect the number of mitochondria (Kan, 2018). After the animal experiment is over, the animal kidney tissue is quickly taken, soaked in glutaraldehyde fixative solution for fixation, and then stored at 4°C for later use. The kidney tissue was embedded in OCT, and frozen section was started after successful embedding. The slices were put in the diluted NAO solution for staining in the dark for 10 min. Finally, the stained slices were mounted with glycerin mounting tablets and observed under a microscope. The fluorescence intensity was calculated with image J software.

### 2.11 Metabolomics analysis

#### 2.11.1 Feces and kidney tissue processing

160 mg feces were weighed and put into an EP tube, added 400 μl of double‐distilled water, and homogenized thoroughly at low temperature. The samples were centrifuge for 15 min (4°C, 21,130 g), and the supernatant was absorbed. 400 μl methanol was added to the remaining residue and homogenized thoroughly at low temperature. The samples were centrifuge for 15 min (4°C, 21,130 *g*), and the supernatant was absorbed. 400 μl acetonitrile was added to the remaining residue and homogenized thoroughly at low temperature. The samples were centrifuged for 15 min (4°C, 21,130 *g*), and the supernatant was absorbed. The above three supernatants were combined, centrifuged for 15 min (4°C, 24,320 *g*). The supernatant was taken for test.

0.2 g kidney tissue was weighed, and 300 μl water and 1.2 ml methanol were added. The samples were homogenized at low temperature, centrifuged for 15 min (4°C, 21,130 *g*), and the supernatant was kept. The supernatant was concentrated by centrifugal concentrator for 3 hr (The vacuum pressure was 0.03). The concentrated sample was reconstituted with 400 μl methanol, vortexed for 5 min, and centrifuged for 15 min (4°C, 21,130 *g*). The supernatant was taken for test.

#### 2.11.2 Chromatographic conditions

Feces sample chromatographic conditions: The UHPLC mobile phase consists of 0.1% formic acid aqueous solution (solvent A) and acetonitrile (solvent B). The steps of gradient elution were as follows: 0–3.0 min, 2%–12% B; 3.0–6.0 min, 12%–31.7% B; 6.0–6.5 min, 31.7%–38.3% B; 6.5–8.5 min, 38.3%–44.9% B; 8.5–12.5 min, 44.9%–51.5% B; 12.5–14.5 min, 51.5%–58.1% B; 14.5–15.5 min, 58.1%–71.3% B; 15.5–20 min, 71.3%–100% B; 20–21 min, 100%–2% B; 21–22 min, 2%–2% B. Kidney tissue sample chromatographic conditions: The UHPLC mobile phase consists of 0.1% formic acid aqueous solution (solvent A) and acetonitrile (solvent B). The steps of gradient elution were as follows: 0–8 min, 2%–28.1% B; 8–10 min, 28.1%–54.1% B; 10–15 min, 54.1%–70.4% B; 15–16 min, 70.4%–80.2% B; 16–18 min, 80.2%–100% B; 18–19 min, 100%–2% B; 19–20 min, 2%–2% B. The temperature of the autosampler and the chromatographic column was maintained at 4 and 35°C, respectively. The flow rate was maintained at 0.4 ml/min.

#### 2.11.3 Mass spectrometry conditions

The mass spectrometer was operated in positive and negative ion mode with Dual electrospray ion source. The positive ion capillary voltage was 4,000 V, and the negative ion capillary voltage was 3,500 V. Atomizer pressure was 30 psig. Drying airflow was 10 L/min. Drying gas temperature was 300°C. Fragmentor voltage was 175 V. Cone voltage was 65 V.

### 2.12 RT‐PCR analysis of Ampk, Sirt1, Pgc‐1α, and Ppar‐α in rat kidney tissues

TRIzol reagent was added to kidney tissue to extract total RNA. GADPH was used as an internal reference for fluorescence quantitative PCR amplification to detect the expression of Ampk, Sirt1, Pgc‐1α, and Ppar‐α related genes in rat kidney tissues of each group. The amplification reaction conditions: 95°C for 10 min, 95°C for 15 s, 60°C for 60 s, 40 cycles in total. The primer sequence was showed in Table 1.

**TABLE 1:** Primer sequence

Gene name	Primer sequence	Fragment length (bp)	Annealing temperature (°C)
R‐SIRT1‐S	AGATTTCAAGGCTGTTGGTTCC	326	60
R‐SIRT1‐A	CAGCATCATCTTCCAAGCCATT		60
R‐AMPK‐S	CACTGGATGCACTCAACACAAC	153	60
R‐AMPK‐A	TCACTACCTTCCATTCAAAGTCC		60
R‐PPAR‐α‐S	GTGGCTGCTATAATTTGCTGTGG	227	60
R‐PPAR‐α‐A	GCGTCTGACTCGGTCTTCTTGA		60
R‐PGC‐1α‐S	TGACCACAAACGATGACCCTC	279	60
R‐PGC‐1α‐A	CTTGGTTGGCTTTATGAGGAGG		60
R‐GADPH‐S	CTGGAGAAACCTGCCAAGTATG	138	60
R‐GADPH‐A	GGTGGAAGAATGGGAGTTGCT		60

### 2.13 Data analysis and processing

PLS‐DA was used to generate molecular formulas of potential biomarkers. Compounds satisfying *p* < .05, *FC* > 2 and *VIP* > 1.0 were selected as biomarkers for preliminary screening. The *M*/*Z* value and retention time of the compound obtained from the analysis were combined with METLIN (http://www.metlin.scipps.edu) and HMDB (www.hmdb.ca) databases to identify the structure of the compound. The identified compound name was input into MetaboAnalyst analysis platform for enrichment and topological analysis, thereby the related metabolic pathways were related screened for potential biomarkers.

All data are expressed as mean ± *SD*. Statistical significance of results was performed with one‐way analysis of variance (ANOVA), using the statistical software SPASS17.0. *p* < .05 was considered statistically significant.

## 3 RESULTS

### 3.1 Effects of XM on ALT, AST, UA, BUN, Cr, TG, TC, TBA, HDL‐C, and LDL‐C

**FIGURE 1:**
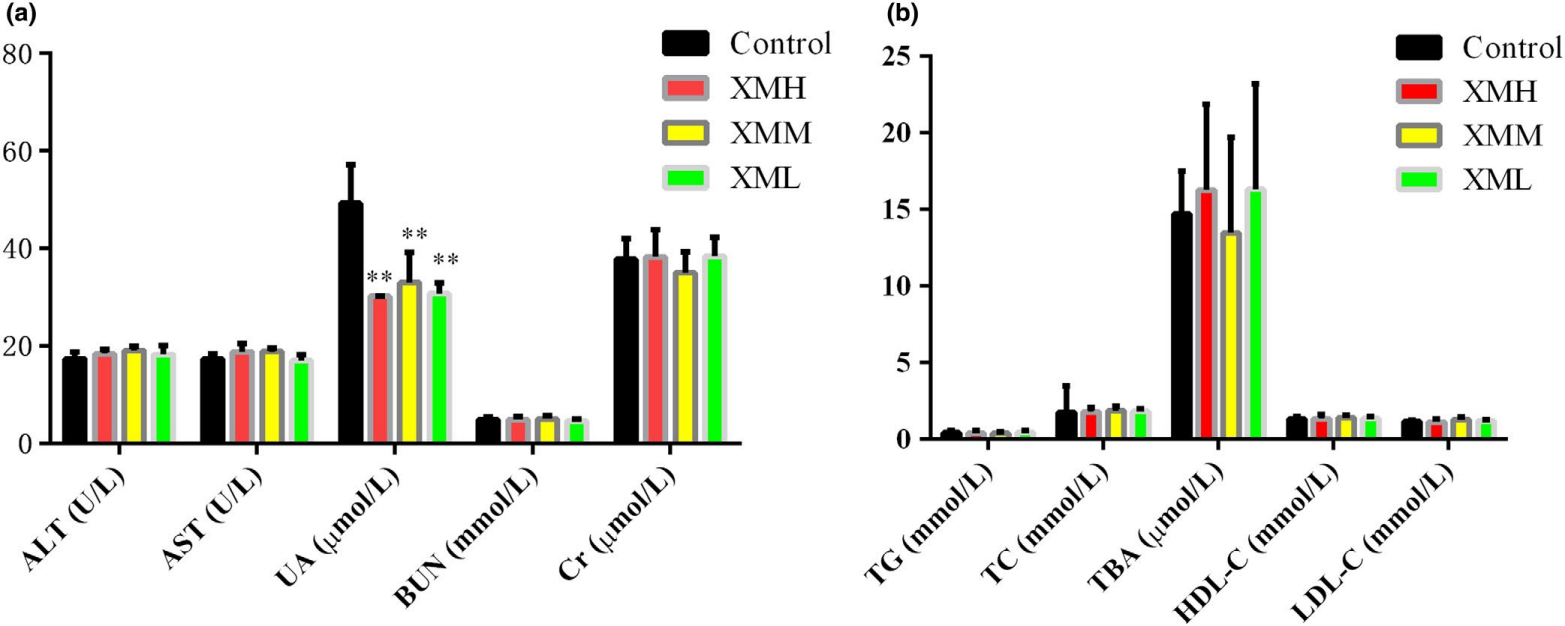
Effects of XM on serum liver, kidney function, and blood lipid in rats

Compared with the blank control group, ALT, AST, BUN, Cr, TG, TC, TBA, HDL‐C, and LDL‐C were not significantly affected by the treatment of XM (*p* > .05), and UA was significantly decreased (*p* < .01) (Figure 1).

### 3.2 Histopathological examination

**FIGURE 2:**
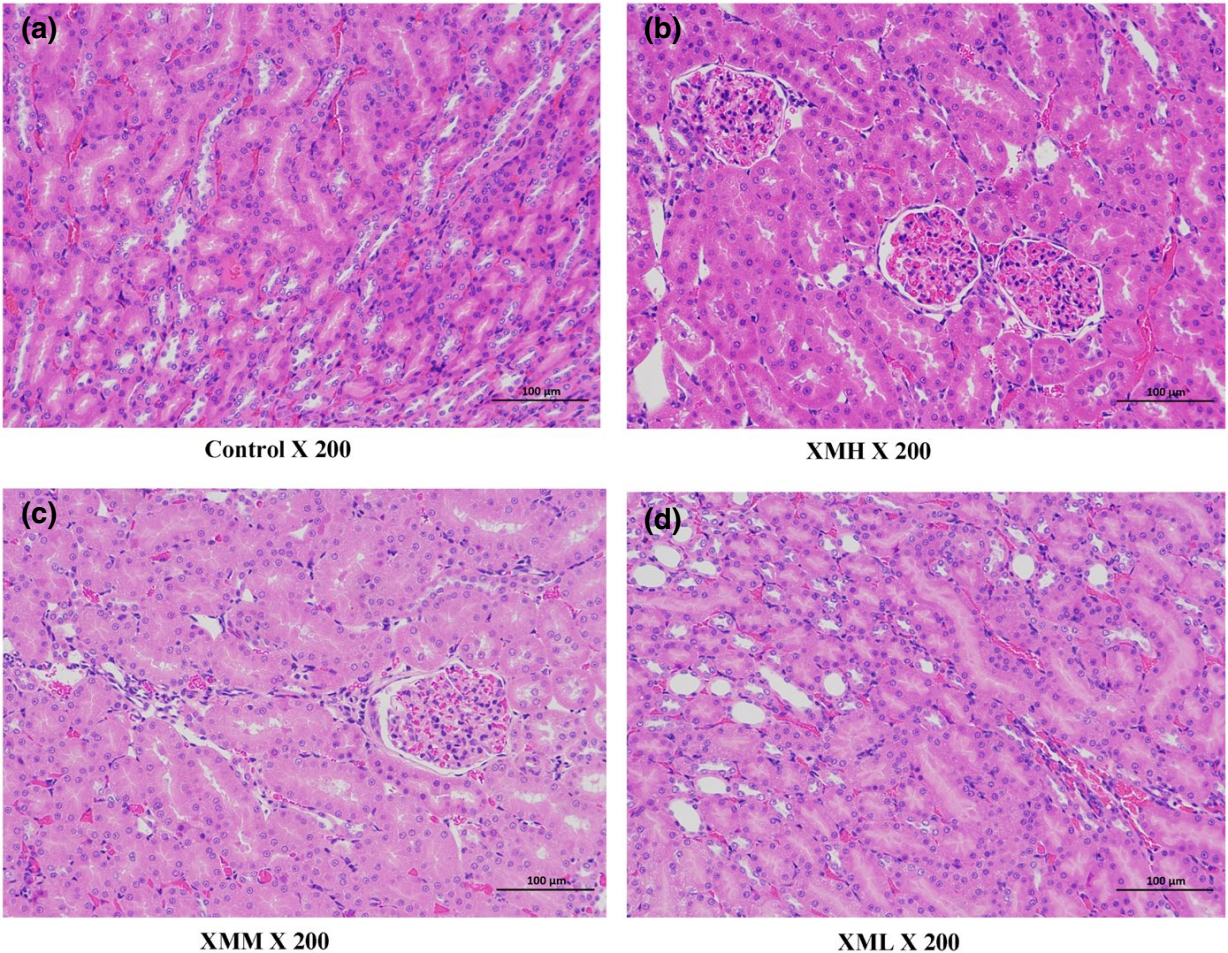
Effects of XM on kidney histopathology in rats

**FIGURE 3:**
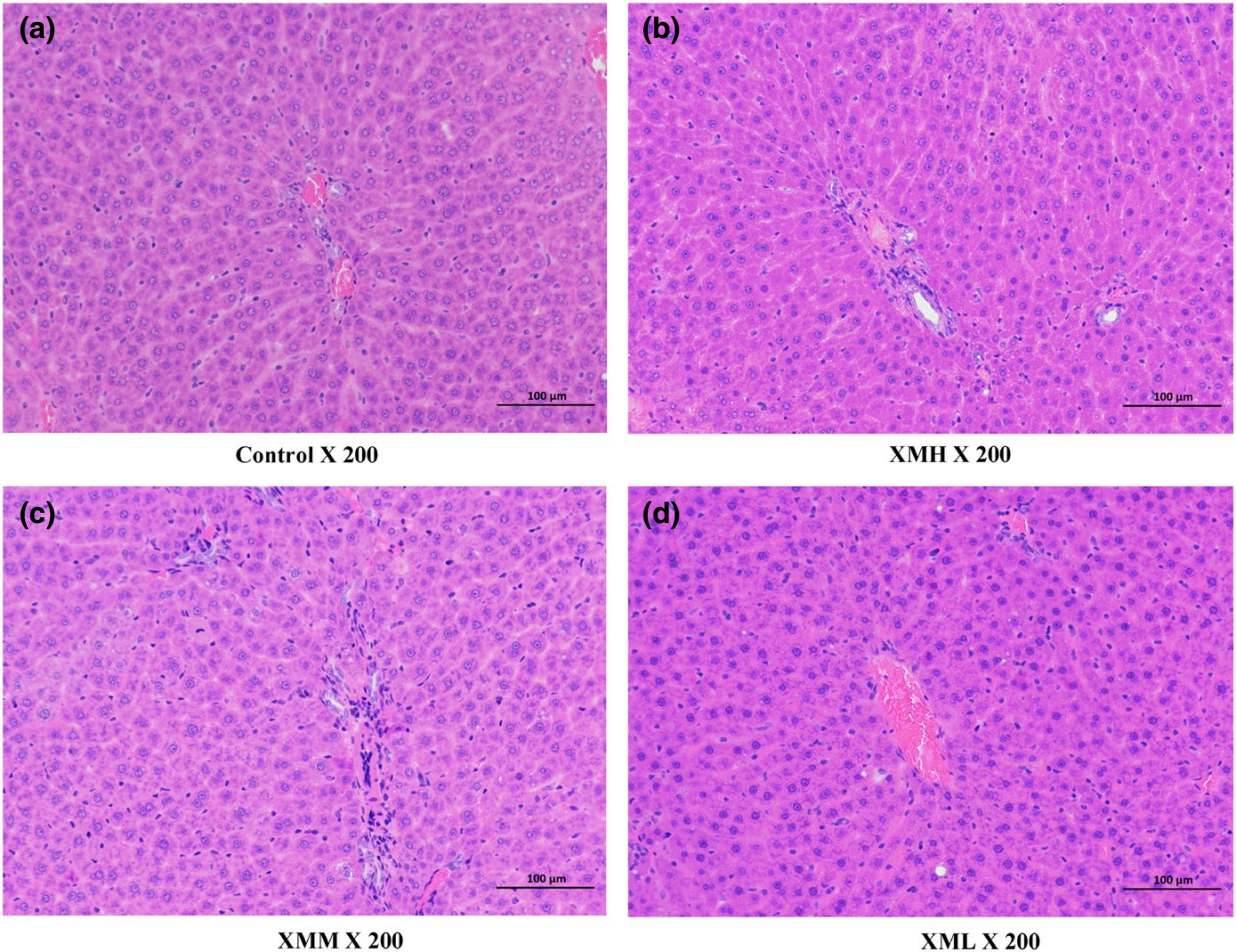
Effects of XM on liver histopathology in rats

Compared with the blank control group, the liver and kidney histopathology of the rats in the XMH, XMM, and XML groups were not abnormal (Figure 2, Figure 3).

### 3.3 Determination of ATP content, Na^+^‐K^+^‐ATPase, Ca^2+^‐Mg^2+^‐ATPase, and SDH enzyme activity in rat kidney tissue

**FIGURE 4:**
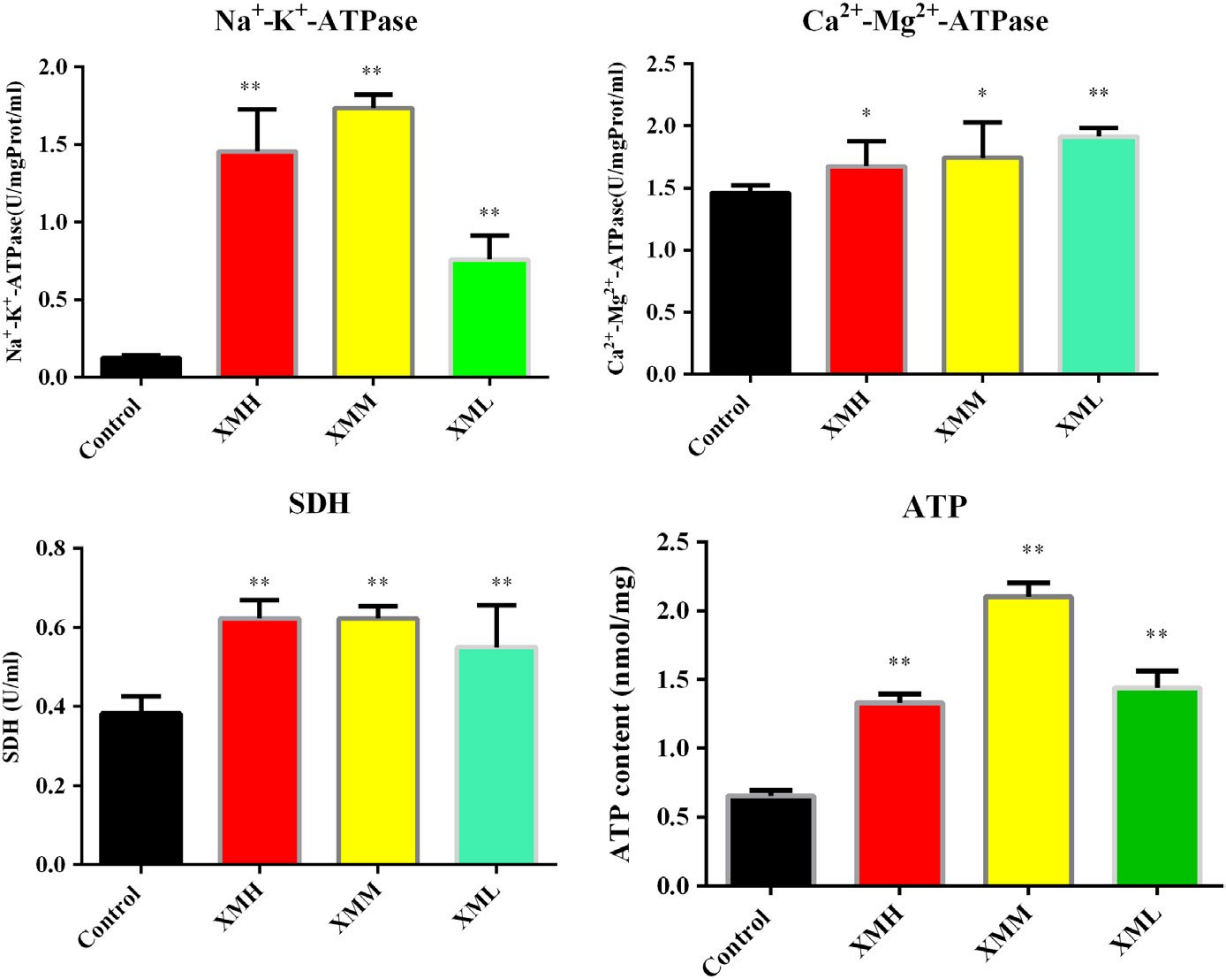
Effects of XM on the activity of ATPase, SDH, and the contents ATP in kidney of rats

Compared with the control group, three different doses of XM all significantly increase the Na^+^‐K^+^‐ATPase, Ca^2+^‐Mg^2+^‐ATPase, and SDH activity in the kidney (*p* < .05, *p* < .01). All three doses of XM can significantly increase the ATP content of kidney tissue (*p* < .01) (Figure 4).

### 3.4 Mitochondrial oxygen consumption rate

**FIGURE 5:**
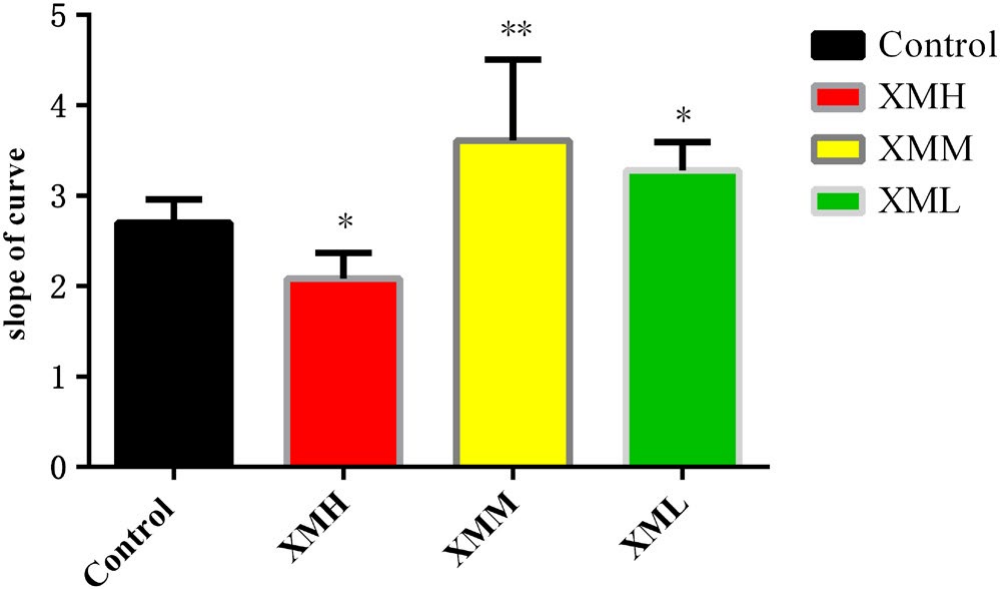
Effects of XM on the metabolic rate of kidney mitochondria in rats

Compared with the blank control group, the slope of the curve in the XMH group decreased significantly (*p* < .05), indicating that the XMH group may inhibit the metabolism of mitochondria. Compared with the blank control group, the slope of the curve of the XMM group and the XML group increased significantly (*p* < .05, *p* < .01), indicating that the mitochondrial metabolism rate increased at these concentration (Figure 5).

### 3.5 NAO fluorescent stain

**FIGURE 6:**
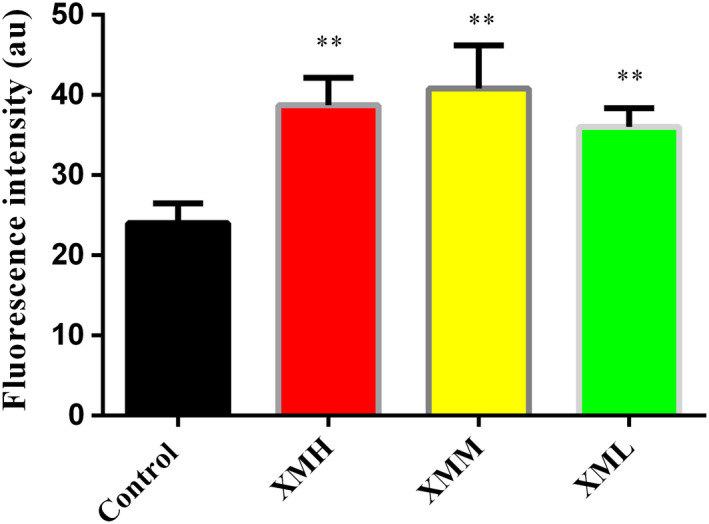
Effect of XM on the fluorescence intensity of kidney mitochondria in rats

**FIGURE 7:**
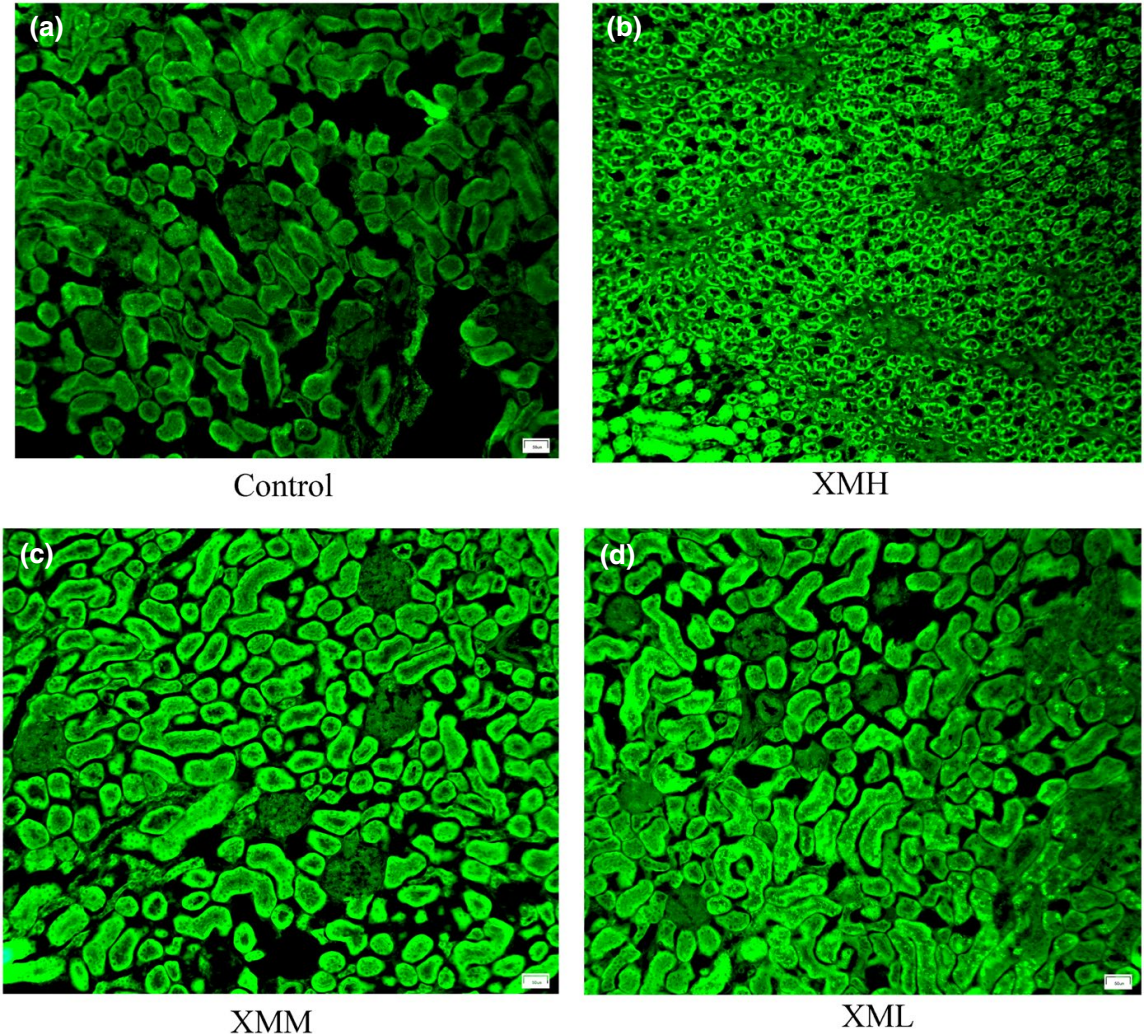
NAO fluorescent stain. (a) Control, (b) XMH, (c) XMM, (d) XML

Compared with the blank control group, the mitochondrial fluorescence intensity was significantly increased in the XMH, XMM, and XML groups (*p* < .01). It suggested that the number of kidney mitochondria increased after the intervention of XM at different concentrations (Figure 6, Figure 7).

### 3.6 Metabolomics analysis

To obtain as much compound information as possible, positive ion and negative ion modes were used for data collection and representative differential metabolites were obtained.

#### 3.6.1 Principal component analysis (PCA)

**FIGURE 8:**
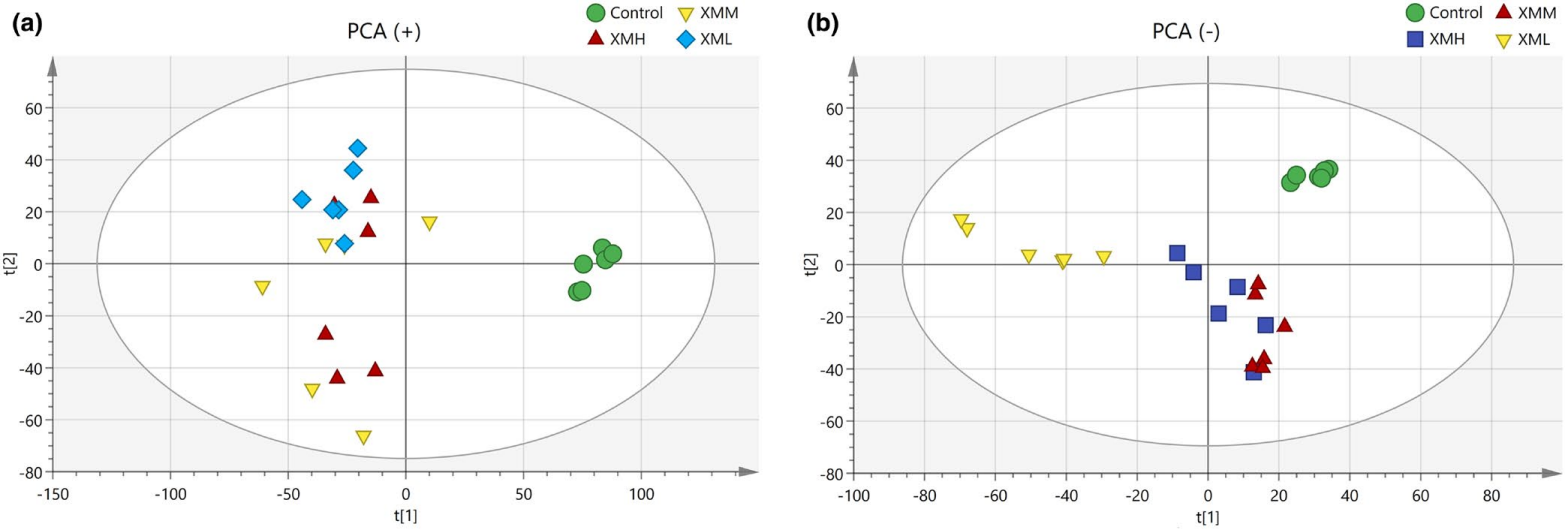
PCA score plot of feces in rats. (a) Positive mode, (b) negative mode

**FIGURE 9:**
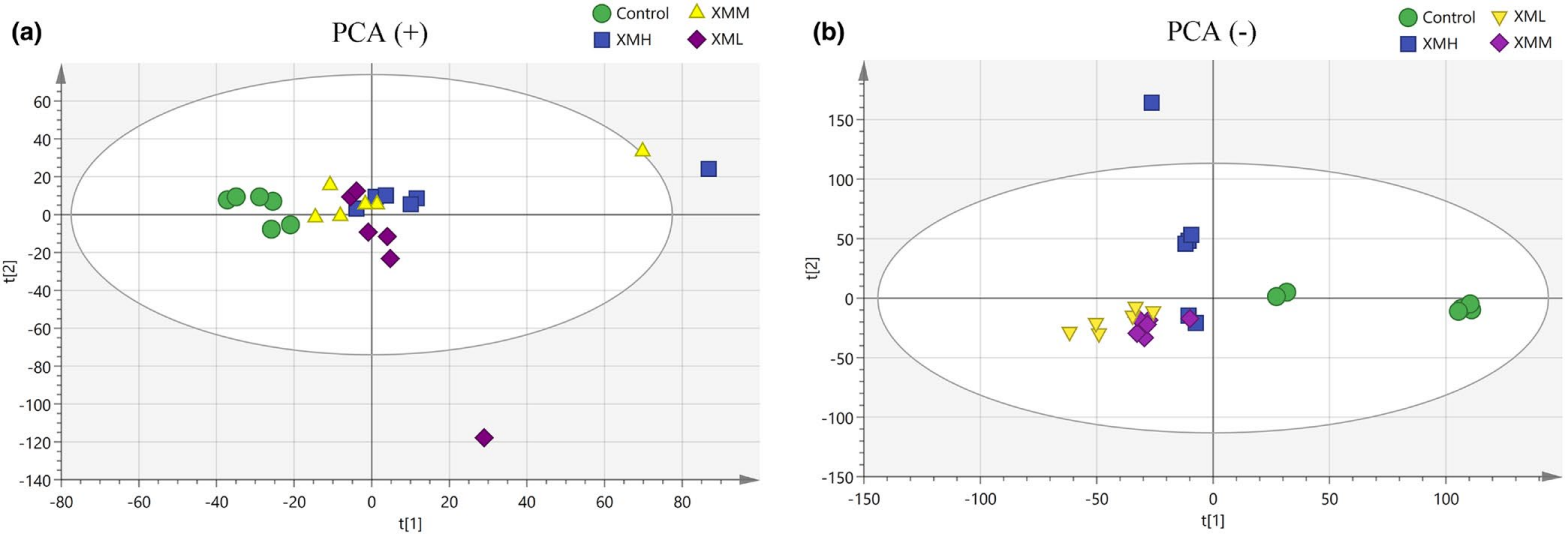
PCA score plot of kidney tissues in rats. (a) Positive mode, (b) negative mode

Metabolic profiles of feces and kidney tissue samples were obtained for each group. In the PCA score plot for feces, principal components showed a *R* ^2^ *X* = 0.526, *Q* ^2^ = 0.0996. As shown in Figure 8, there was clear separation among rats in the XMH, XMM, and XML. In the PCA score plot for kidney tissues, principal components showed a *R* ^2^ *X* = 0.59, *Q* ^2^ = 0.228. As shown in Figure 9, there was clear separation among rats in the XMH, XMM, and XML, but there were greater variation and more discrete aggregation, suggesting that the effect of XM on individual rats was highly variable. PCA could be used for a preliminary assessment of the metabolite profiles of rats in different groups; however, owing to the variation within groups (and inability to highlight the differences between groups), further PLS‐DA was needed.

#### 3.6.2 Partial least squares discrimination analysis (PLS‐DA) of feces in different groups

**FIGURE 10:**
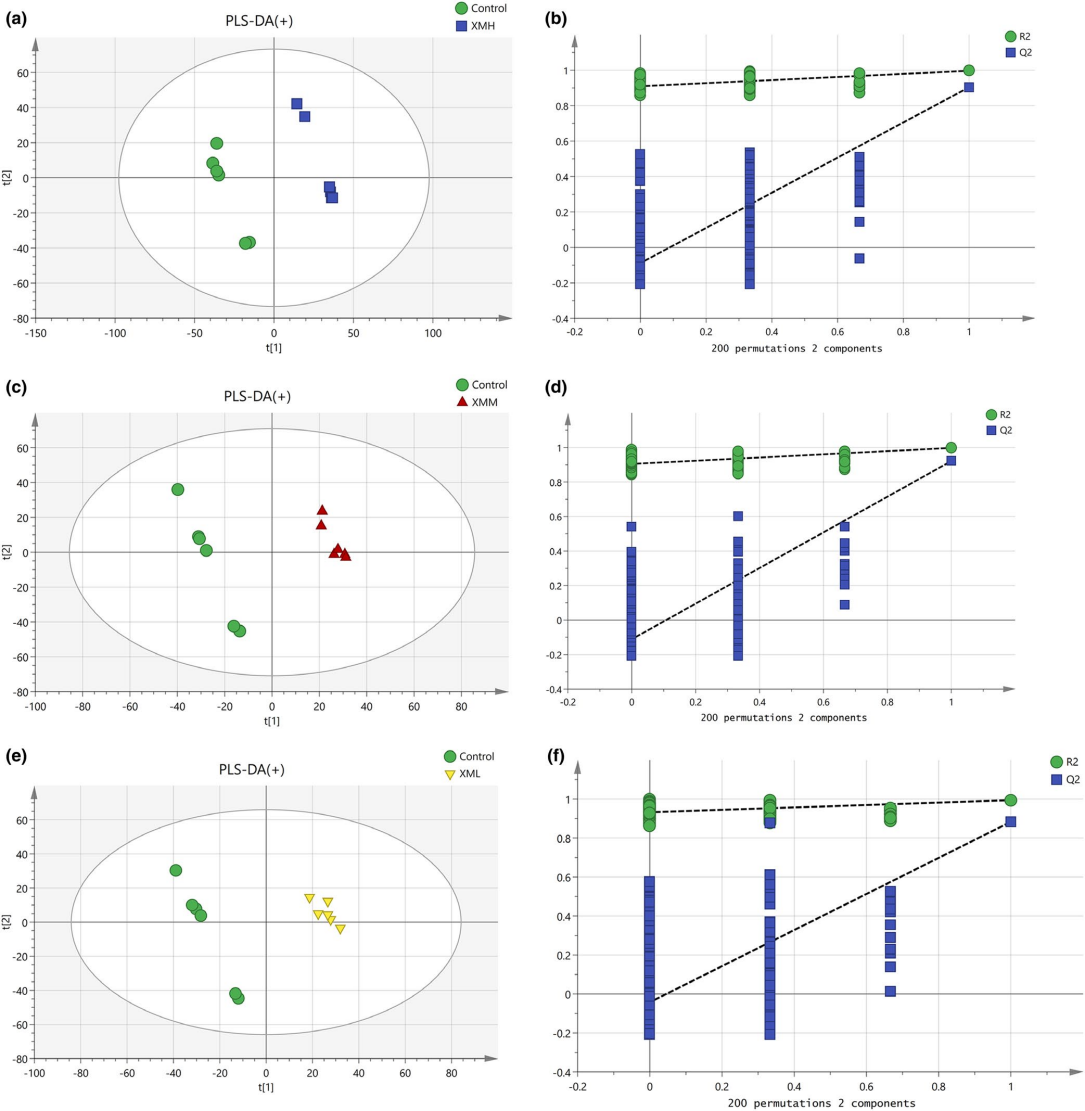
PLS‐DA analysis and permutation test of feces in the positive mode. (a) PLS‐DA score plot of XMH and Control groups, (b) permutation test of XMH and Control groups, (c) PLS‐DA score plot of XMM and Control groups, (d) Permutation test of XMM and Control groups, (e) PLS‐DA score plot of XML and Control groups, (f) permutation test of XML and Control groups

**FIGURE 11:**
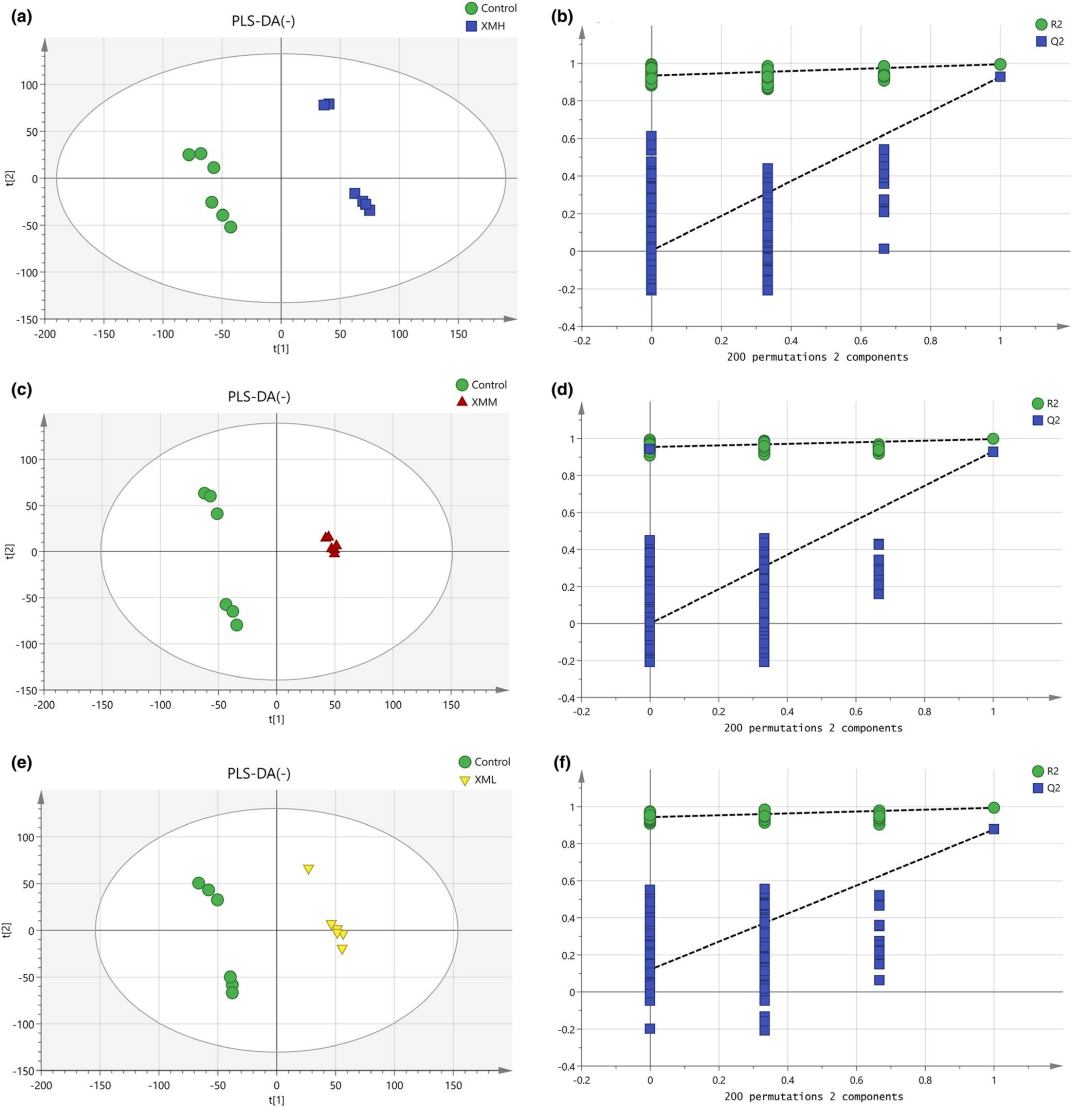
PLS‐DA analysis and permutation test of feces in the negative mode. (a) PLS‐DA score plot of XMH and Control groups, (b) Permutation test of XMH and Control groups, (c) PLS‐DA score plot of XMM and Control groups, (d) permutation test of XMM and Control groups, (e) PLS‐DA score plot of XML and Control groups, (f) permutation test of XML and Control groups

As shown in Figure 10a, an PLS‐DA mode for feces in the XMH and Control was established in the positive mode. The samples of feces in the XMH and Control groups were completely spatially separated, with *R* ^2^ *X* = 0.513, *R* ^2^ *Y* = 0.997, *Q* ^2^ = 0.904, indicating that the mode had good predictive ability. As shown in Figure 11a, an PLS‐DA mode for feces in the XMH and Control was established in the negative mode. The samples of feces in the XMH and Control were completely spatially separated, with *R* ^2^ *X* = 0.474, *R* ^2^ *Y* = 0.995, *Q* ^2^ = 0.928, indicating that the mode had good predictive ability.

As shown in Figure 10c, an PLS‐DA mode for feces in the XMM and Control was established in the positive mode. Samples of feces in the XMM and Control groups were completely spatially separated, with *R* ^2^ *X* = 0.495, *R* ^2^ *Y* = 0.998, *Q* ^2^ = 0.921, indicating that the mode had good predictive ability. As shown in Figure 11c, an PLS‐DA mode for feces in the XMM and Control was established in the negative mode. Samples of feces in the XMM and Control groups were completely spatially separated, with *R* ^2^ *X* = 0.494, *R* ^2^ *Y* = 0.997, *Q* ^2^ = 0.931, indicating that the mode had good predictive ability.

As shown in Figure 10e, an PLS‐DA mode for feces in the XML and Control was established in the positive mode. Samples of feces in the XML and Control groups were completely spatially separated, with *R* ^2^ *X* = 0.506, *R* ^2^ *Y* = 0.994, *Q* ^2^ = 0.883, indicating that the mode had good predictive ability. As shown in Figure 11e, an PLS‐DA mode for feces in the XML and Control was established in the negative mode. Samples of feces in the XML and Control groups were completely spatially separated, with *R* ^2^ *X* = 0.531, *R* ^2^ *Y* = 0.994, *Q* ^2^ = 0.877, indicating that the mode had good predictive ability.

A permutation test repeated 200 times, as shown in Figures 10b,d,f and 11b,d,f showed that as *Y* variables increased, *R* ^2^ and *Q* ^2^ gradually declined, indicated that the mode was highly robust and there was no overfitting.

#### 3.6.3 PLS‐DA of kidney tissues in different groups

**FIGURE 12:**
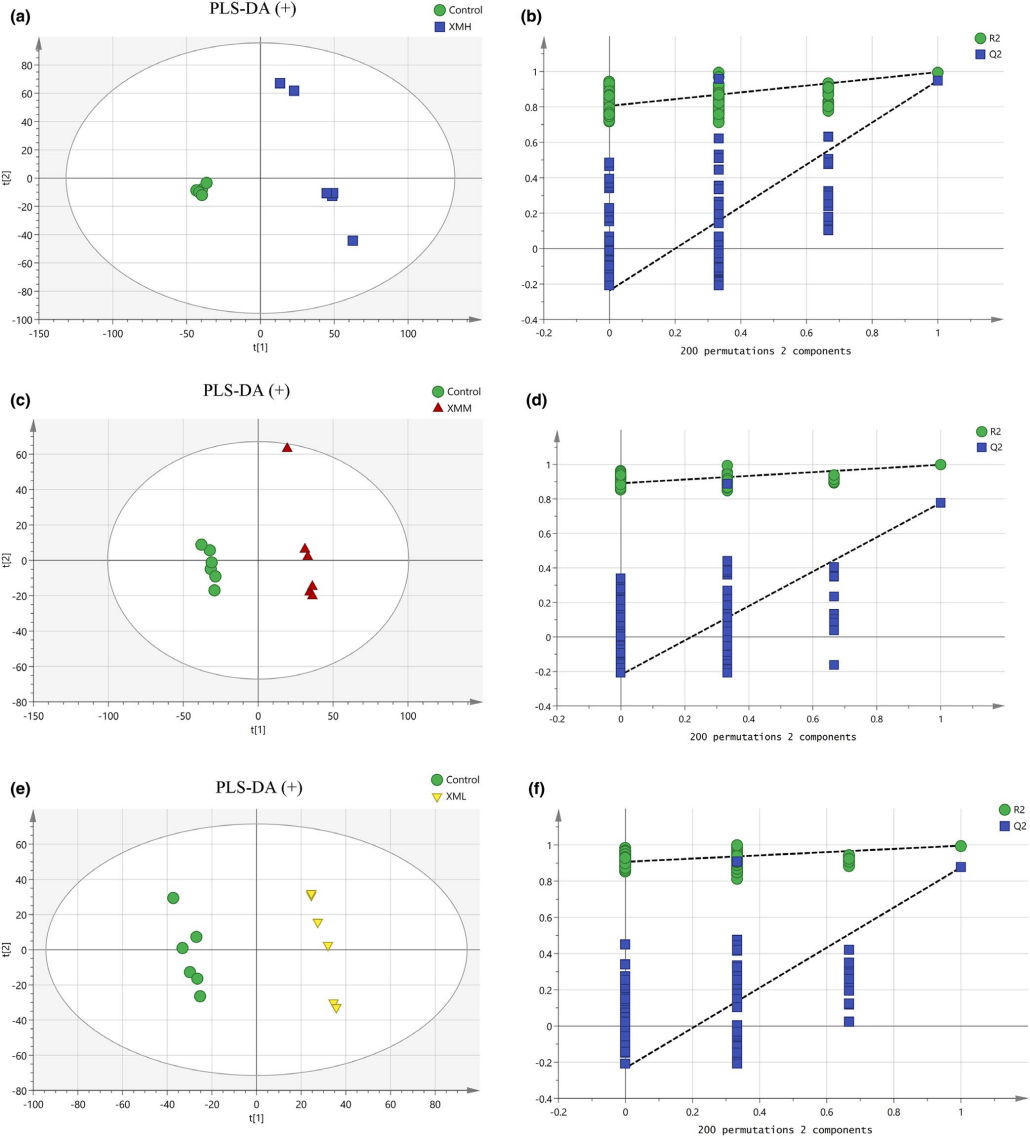
PLS‐DA analysis and permutation test of kidney tissues in the positive mode. (a) PLS‐DA score plot of XMH and Control groups, (b) permutation test of XMH and Control groups, (c) PLS‐DA score plot of XMM and Control groups, (d) permutation test of XMM and Control groups, (e) PLS‐DA score plot of XML and Control groups, (f) permutation test of XML and Control groups

**FIGURE 13:**
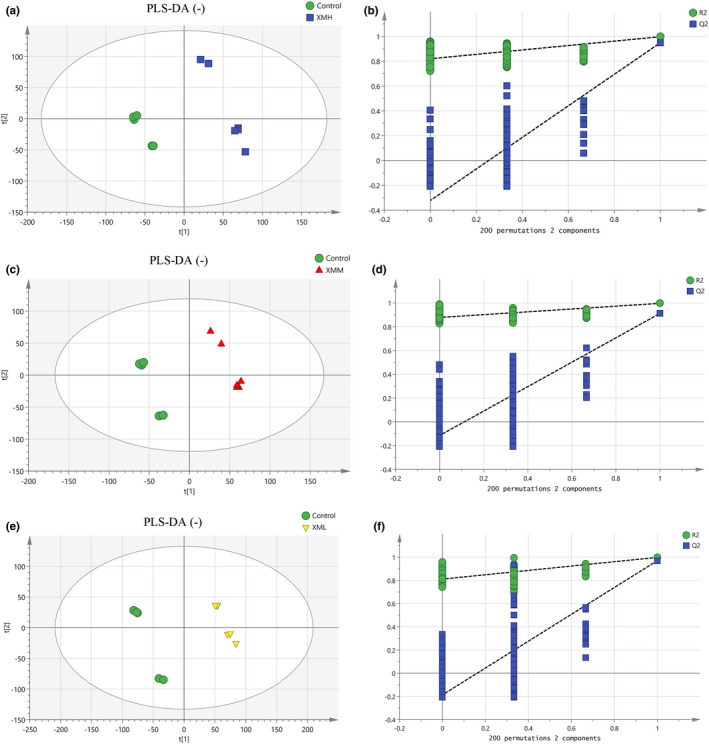
PLS‐DA analysis and permutation test of kidney tissues in the negative mode. (a) PLS‐DA score plot of XMH and Control groups, (b) permutation test of XMH and Control groups, (c) PLS‐DA score plot of XMM and Control groups, (d) permutation test of XMM and Control groups, (e) PLS‐DA score plot of XML and Control groups, (f) permutation test of XML and Control groups

As shown in Figure 12a, an PLS‐DA mode for kidney tissues in the XMH and Control was established in the positive mode. The samples of kidney tissues in the XMH and Control groups were completely spatially separated, with *R* ^2^ *X* = 0.538, *R* ^2^ *Y* = 0.996, *Q* ^2^ = 0.949, indicating that the mode had good predictive ability. As shown in Figure 13a, an PLS‐DA mode for kidney tissues in the XMH and Control was established in the negative mode. The samples of kidney in the XMH and Control groups were completely spatially separated, with *R* ^2^ *X* = 0.607, *R* ^2^ *Y* = 0.997, *Q* ^2^ = 0.971, indicating that the mode had good predictive ability.

As shown in Figure 12c, an PLS‐DA mode for kidney tissues in the XMM and Control was established in the positive mode. Samples of kidney tissues in the XMM and Control groups were completely spatially separated, with *R* ^2^ *X* = 0.563, *R* ^2^ *Y* = 0.998, *Q* ^2^ = 0.778, indicating that the mode had good predictive ability. As shown in Figure 13c, an PLS‐DA mode for kidney in the XMM and Control was established in the negative mode. Samples of kidney tissues in the XMM and Control groups were completely spatially separated, with *R* ^2^ *X* = 0.547, *R* ^2^ *Y* = 0.997, *Q* ^2^ = 0.912, indicating that the mode had good predictive ability.

As shown in Figure 12e, an PLS‐DA mode for kidney tissues in the XML and Control was established in the positive mode. Samples of kidney tissues in the XML and Control groups were completely spatially separated, with *R* ^2^ *X* = 0.494, *R* ^2^ *Y* = 0.998, *Q* ^2^ = 0.888, indicating that the mode had good predictive ability. As shown in Figure 13e, an PLS‐DA mode for kidney tissues in the XML and Control was established in the negative mode. Samples of kidney tissues in the XML and Control group were completely spatially separated, with *R* ^2^ *X* = 0.491, *R* ^2^ *Y* = 0.998, *Q* ^2^ = 0.970, indicating that the mode had good predictive ability.

A permutation test repeated 200 times, as shown in Figures 12b,d,f and 13b,d,f showed that as *Y* variables increased, *R* ^2^ and *Q* ^2^ gradually declined, indicated that the mode was highly robust and there was no overfitting.

#### 3.6.4 Potential biomarkers in feces and kidney tissues

**FIGURE 14:**
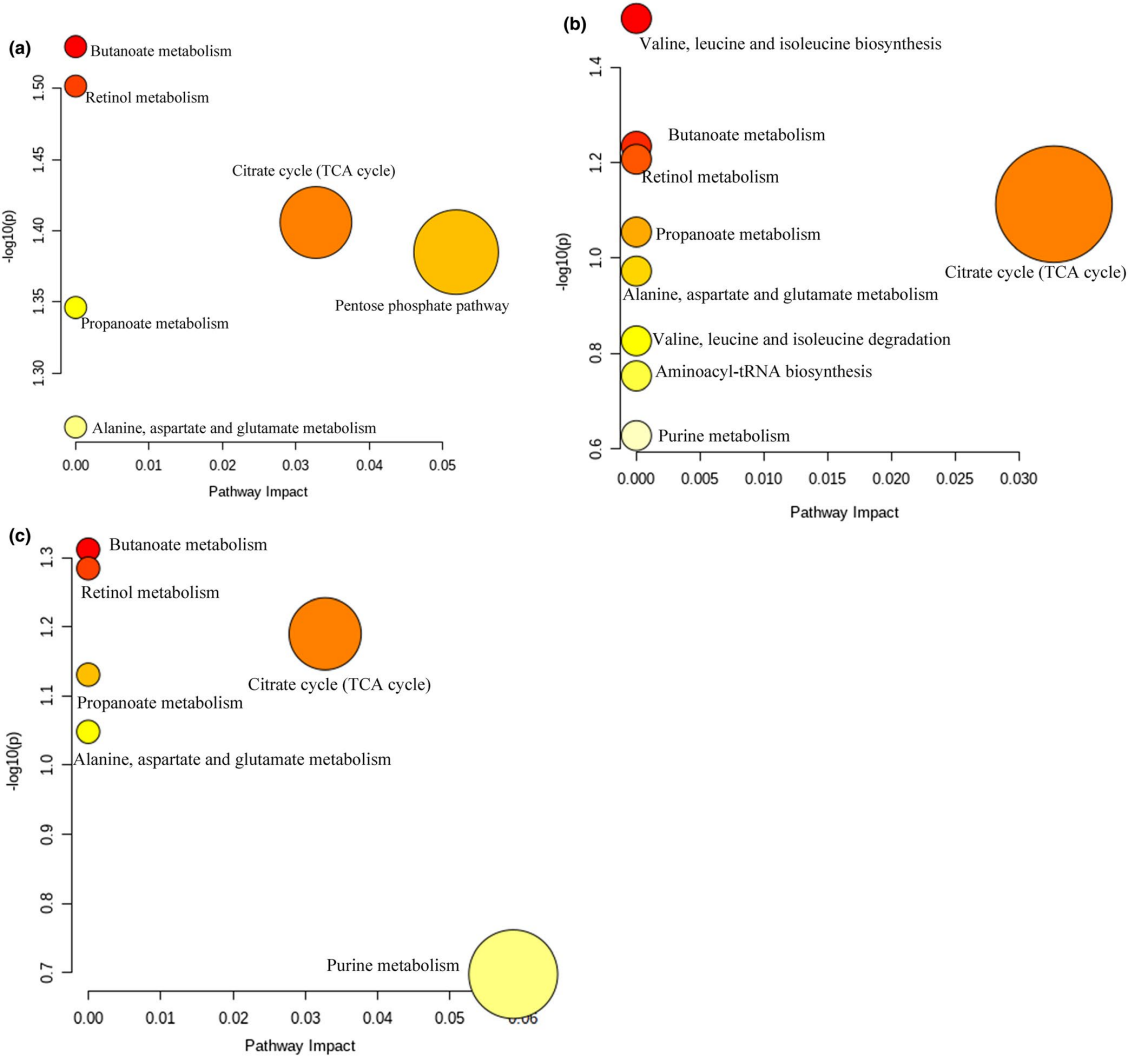
Potential biomarkers pathway analysis of rats. (a) XMH group, (b) XMM group, (c) XML group

The result showed that eight potential biomarkers were identified from XMH (Table 2). They were 2‐glycerolphosphatecholinearachidonicacid, ceanothenic acid, linoelaidic acid, 11‐cis‐retinaldehyde, katonic acid, levulinic acid, succinic acid, and gluconic acid. The contents of gluconic acid, 2‐glycerolphosphatecholinearachidonicacid, and ceanothenic acid were decreased. The contents of linoelaidic acid, 11‐cis‐retinaldehyde, katonic acid, levulinic acid, and succinic acid were increased. As shown in Figure 14a, six metabolic pathways were screened between XMH and the blank control group, mainly including butanoate metabolism, retinol metabolism, citrate cycle (TCA cycle), pentose phosphate pathway, propanoate metabolism, alanine, aspartate, and glutamate metabolism.

**TABLE 2:** Potential biomarkers of feces and tissues in the XMH

Potential biomarkers	*M*/*Z*	Molecular formula	RT	Up/Down
Feces				
2‐glycerolphosphatecholine arachidonic acid	544.3401	C_28_H_50_NO_7_P	13.254	Down
Ceanothenic acid	455.3127	C_29_H_42_O_4_	16.628	Down
Linoelaidic acid	281.2468	C_18_H_32_O_2_	9.463	Up
11‐cis‐Retinaldehyde	285.2209	C_20_H_28_O	12.235	Up
Katonic acid	457.3668	C_30_H_48_O_3_	14.206	Up
Levulinic acid	115.0401	C_5_H_8_O_3_	5.655	Up
Kidney tissue				
Succinic acid	117.0192	C_4_H_6_O_4_	1.101	Up
Gluconic acid	195.0506	C_6_H_12_O_7_	1.029	Down

Table 3 showed that eight potential biomarkers were identified from XMM. They were katonic acid, oleamide, 11‐cis‐retinaldehyde, L‐Leucine, 2‐keto‐3‐deoxy‐D‐gluconic acid, xanthosine, succinic acid, and tauroursodeoxycholic acid. The content of L‐Leucine, 2‐Keto‐3‐deoxy‐D‐gluconic acid, and xanthosine was decreased. The content of katonic acid, oleamide, 11‐cis‐Retinaldehyde, succinic acid, and tauroursodeoxycholic acid was increased. As shown in Figure 14b, nine metabolic pathways were screened between XMM and the blank control group, mainly including valine, leucine, and isoleucine biosynthesis, butanoate metabolism, retinol metabolism, citrate cycle (TCA cycle), propanoate metabolism, alanine, aspartate, and glutamate metabolism, valine, leucine, and isoleucine degradation, aminoacyl‐tRNA biosynthesis, and purine metabolism.

**TABLE 3:** Potential biomarkers of feces and tissues in the XMM

Potential biomarkers	*M*/*Z*	molecular formula	RT	Up/Down
Feces				
Katonic acid	457.3668	C_30_H_48_O_3_	14.206	Up
Oleamide	282.2788	C_18_H_35_NO	9.346	Up
11‐cis‐Retinaldehyde	285.2209	C_20_H_28_O	12.235	Up
L‐Leucine	130.0873	C_6_H_13_NO_2_	0.795	Down
2‐Keto‐3‐deoxy‐D‐gluconic acid	177.0405	C_6_H_10_O_6_	0.661	Down
Kidney tissue				
Xanthosine	285.0830	C_10_H_12_N_4_O_6_	1.789	Down
Succinic acid	117.0192	C_4_H_6_O_4_	1.101	Up
Tauroursodeoxycholic acid	498.2888	C_26_H_45_NO_6_S	10.866	Up

Table 4 showed that ten potential biomarkers were identified from XML. They were nutriacholic acid, 2‐glycerolphosphatecholinearachidonicacid, 11‐cis‐retinaldehyde, katonic acid, ceanothenic acid, maleylacetic acid, isoleucylproline, LysoPE (18:0/0:0), succinic acid, and adenosine monophosphate. The content of 2‐glycerolphosphatecholinearachidonicacid, ceanothenic acid, maleylacetic acid, isoleucylproline, and LysoPE (18:0/0:0) was decreased. The content of nutriacholic acid, 11‐cis‐retinaldehyde, katonic acid, succinic acid, and adenosine monophosphate was increased. As shown in Figure 14c, six metabolic pathways were screened between XML and the blank control group, mainly including butanoate metabolism, retinol metabolism, citrate cycle (TCA cycle), propanoate metabolism, alanine, aspartate, and glutamate metabolism, and purine metabolism.

**TABLE 4:** Potential biomarkers of feces and tissues in the XML

Potential biomarkers	*M*/*Z*	molecular formula	RT	Up/Down
Feces				
Nutriacholic acid	391.284	C_24_H_38_O_4_	9.597	Up
2‐glycerolphosphatecholine arachidonic acid	544.3401	C_28_H_50_NO_7_P	13.254	Down
11‐cis‐Retinaldehyde	285.2209	C_20_H_28_O	12.235	Up
Katonic acid	457.3668	C_30_H_48_O_3_	14.206	Up
Ceanothenic acid	455.3127	C_29_H_42_O_4_	16.628	Down
Kidney tissue				
Maleylacetic acid	159.0281	C_6_H_6_O_5_	4.461	Down
Isoleucylproline	229.1539	C_11_H_20_N_2_O_3_	2.774	Down
LysoPE (18:0/0:0)	482.3235	C_23_H_48_NO_7_P	11.726	Down
Succinic acid	117.0192	C_4_H_6_O_4_	1.101	Up
Adenosine monophosphate	346.0563	C_10_H_14_N_5_O_7_P	1.329	Up

### 3.7 RT‐PCR

**FIGURE 15:**
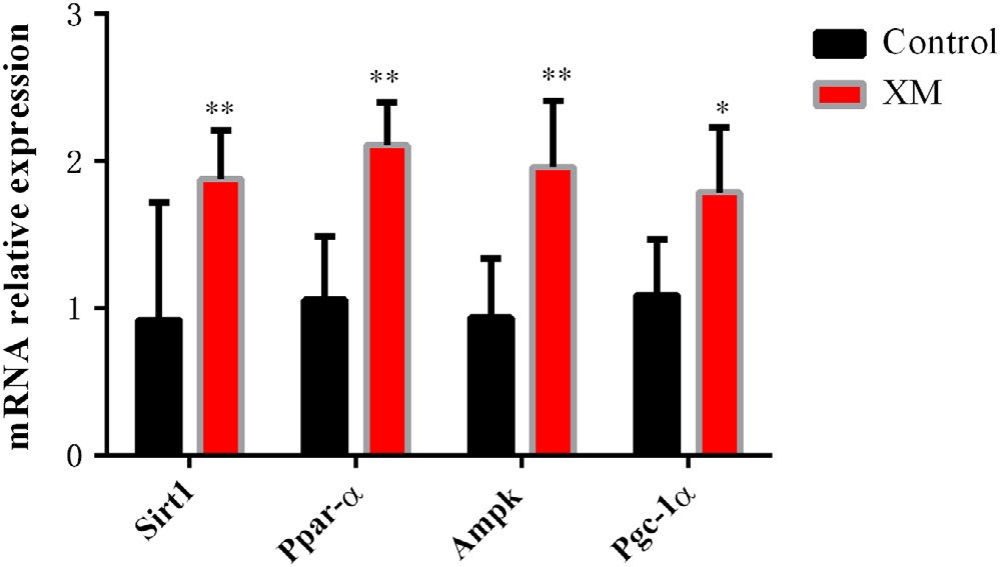
Effects of XM on Ampk, Sirt1, Pgc‐1α, and Ppar‐α

The above results showed that high, medium, and low doses of XM could promote kidney energy metabolism in rats, so we chose one of the dose groups (chose medium dose of XM) to carry out the experimental verification of its upstream mRNA related to energy metabolism. RT‐PCR results showed that the relative expression levels of Ampk, Sirt1, Pgc‐1α, and Ppar‐α in XM group were significantly higher than those in the blank control group (*p* < .05, *p* < .01) (Figure 15).

## 4 DISCUSSION

### 4.1 Effects of different doses of XM on liver and kidney function in rats

When the high dose of XM extract is 15 g/kg and the low dose is 5 g/kg, continuous administration for 60 days would cause certain adverse reactions to the physiological and biochemical functions of the liver (Chen, 2011). It was found that when the dosage was 100 times of the clinical dosage, the long‐term administration for 3 months showed no obvious toxicity to the liver and kidney function (Xiang et al., 2006; Zhang et al., 2005). When the alcohol extract of XM was administered continuously for 30 days at a dose of 120 g/kg, there was no significant difference in serum urea nitrogen, creatinine, and alanine aminotransferase, and after 90 days of continuous administration, serum urinary nitrogen, creatinine, and alanine aminotransferase increased significantly, but there was no significant change in liver and kidney organs (Bao et al., 2011). Long‐term super dose administration of XM may cause accumulation toxicity, resulting in physiological and pathological changes of liver and kidney, and the toxicity of alcohol extract of XM was significantly higher than that of water extract of XM (Bao et al., 2011). In this study, the medium dose for rats was converted from the usual human dose in the Chinese Pharmacopoeia. After the medium dose was determined, the high and low doses were determined according to the principle of pharmacological dose. The results of this study showed that XM water extract had no significant in serum liver and kidney indicators, blood lipids, and liver and kidney organs. It indicated that XM had no effect on liver and kidney function at the dose of this experiment. This may be related to the dosage, time of administration, and extraction solvent of XM.

### 4.2 Effects of different doses of XM on the activities of ATPase, SDH enzyme, and ATP content in rats

ATPase exists on the cell membrane of tissue cells and organelles. It is a kind of protease on biological membrane, which plays an important role in material transmission, energy conversion, information transmission, etc. (Sudar et al., 2008). Na^+^‐K^+^‐ATPase is a complex membrane protein, which can use the energy produced by ATP hydrolysis to transport three Na^+^ ions out of the cell and transfer two K^+^ ions into the cell at the same time. This enzyme produces a lift on the cell membrane to maintain the resting potential of the cell (Ma et al., 2019). The Na^+^ ion gradient drives many transport processes through cotransportation (such as glucose cotransporter), exchangers (Na^+^/Ca^2+^ exchanger), and drives amino acids and vitamins into cells (Lingrel, 2010). It was found that when the activity of Na^+^‐K^+^‐ATPase decreased, the amount of Na^+^ ions transported out of the cell decreased, which would lead to intracellular Na^+^ overload, and then activate the Na^+^‐Ca^2+^ exchange protein on the membrane, At the same time, it would also lead to the release of calcium ions in mitochondria, which would lead to intracellular Ca^2+^ overload, and the decrease in Mg^2+^ activity will not only reduce the content of Mg^2+^ in cells, but also reduce the activities of other enzymes related to energy metabolism and inhibit energy generation (Bao et al., 2004). Therefore, when the activities of Na^+^‐K^+^‐ATPase and Ca^2+^‐Mg^2+^‐ ATPase are inhibited, it not only affects the hydrolysis of ATP, but also leads to the obstacle of intracellular ion transport, aggravating the obstacle of energy supply and application (Zhai et al., 2016). ATP is the energy material directly used by the body, and its content can reflect the ability of mitochondrial oxidative phosphorylation and the state of energy generation. Succinate dehydrogenase has dual functions, that is, it plays an important role in both the tricarboxylic acid cycle and the aerobic respiratory chain, catalyzing the oxidation of succinate to fumarate and the reduction of penguinone to quinone (Acevedo et al., 2013), converting succinic acid to fumarate, dehydrogenating FDAH, and then oxidizing FDA transmitter to generate energy (Wang, Jia, et al., 2020). The decrease in SDH activity will inhibit TCA cycle and oxidative phosphorylation, lead to mitochondrial dysfunction, and ultimately affect the efficiency of energy synthesis (Ekekcioglu et al., 1999). This study results showed that the activities of Na^+^‐K^+^‐ATPase, Ca^2+^‐Mg^2+^‐ATPase, and SDH were increased in high, medium, and low doses of XM groups, which indicated that XM could promote the activity of ATPase, increase energy consumption, and increase energy production by strengthening TCA cycle, thus promoting energy metabolism. The content of ATP increased in high, medium, and low dose groups, which indicated that in the experimental conditions, the energy production of rats may be greater than energy consumption.

### 4.3 Effects of different doses of XM on mitochondrial metabolic rate and mitochondrial number in rats

Mitochondria are the main sites for ATP synthesis, and more than 80% of the energy required for life activities comes from mitochondria (Liu et al., 1988). It is found that low dose aconite can promote the metabolism of mitochondria, and when the dose reaches a certain concentration, the metabolism of mitochondria will be inhibited (Zheng, 2015), indicating that the drug dose may affect the metabolic rate of mitochondria. This study results showed that the high dose of XM group decreased the mitochondrial oxygen consumption rate, while the medium dose of XM group and low dose of XM group increased the mitochondrial oxygen consumption rate in varying degrees, suggesting that the mitochondrial metabolic rate may be related to the dosage. From the results of mitochondrial number, different doses of XM increased the number of mitochondria in varying degrees, indicating that high dose of XM may increase the number of mitochondria to increase the body's energy, while medium dose and low dose of XM can regulate the body's partial energy metabolism by increasing the metabolic rate and the number of mitochondria.

### 4.4 Analysis of metabolic pathway

#### 4.4.1 TCA cycle, butanoate metabolism, propanoate metabolism, alanine, aspartate, and glutamate metabolism

In this experimental condition, the high, medium, and low doses of XM groups were enriched the TCA cycle, butanoate, propanoate metabolism, alanine, aspartate, and glutamate metabolism, and the involved differential metabolism was succinic acid in which the content was increased. TCA cycle is an important pathway of aerobic catabolism of sugar, fat, and amino acids, in which intermediate products are the initiation of many biosynthetic pathways (Peng et al., 2010). The state of TCA cycle can reflect the state of energy metabolism. Succinic acid is an intermediate product of the TCA cycle. It forms corydalis under the action of succinate dehydrogenase and continues to participate in the next step of the TCA cycle. Combined with the biochemical results of succinate dehydrogenase activity, it is speculated that XM may promote kidney energy synthesis by increasing succinate in TCA cycle pathway.

#### 4.4.2 Retinol metabolism

Retinol is an important cofactor in activating mitochondrial protein kinase C (PKC) (Kim & Hammerling, 2020), which activates PKC through redox. After the activation of PKC, it can increase the production of acetyl CoA, then stimulates PDH (pyruvate dehydrogenase) complex (Patel & Korotchinka, 2006; Patel & Roche, 1990), and then increase the utilization rate of pyruvate (Acin‐Perez et al., 2010), promoting oxidative phosphorylation in mitochondria, resulting in increased oxygen consumption and ATP synthesis in mitochondria. In addition, retinol can be used as the carrier of mitochondrial electron, which can accelerate the electron transfer after binding with PKC, and then promote the process of oxidative phosphorylation (Hammerling, 2016). In the conditions of this experiment, the high, medium, and low doses of XM groups were enriched in the retinol metabolic pathway, and the related differential metabolite was retinaldehyde in which the content was increased. Retinol can be converted to retinaldehyde after oxidation, which indicates that retinol may be oxidized to retinaldehyde in the process of activating mitochondrial protein kinase or acting as mitochondrial electron transport carrier.

#### 4.4.3 Valine, leucine, and isoleucine, aminoacyl‐tRNA biosynthesis metabolism

Leucine and isoleucine belong to branched chain amino acids (BCCA) and are potential nutritional signaling molecules. Leucine can regulate glucose metabolism, fat synthesis, and decomposition, improve insulin sensitivity, and promote energy metabolism (Higashi et al., 2015; Wang et al., 2011; Xu et al., 2015). In healthy rats, absorption and oxidation of branched chain amino acids occur in many different tissues, Once it enters cells, BCAA can be stored in amino acid pools, integrated into proteins, or sent to mitochondria for oxidation, and can also be used for the synthesis of ketones and glucose (Neinast et al., 2019). On the one hand, BCAA transfers amino groups from BCAAs (branched chain α‐ketoglutarate) to α‐ketoglutarate to form glutamate under the catalysis of BCAT (branched chain transaminase) and then combines with pyruvate to form alanine through alanine aminotransferase, which participates in TCA cycle. The level of BCAA can affect the intermediates of TCA (tricarboxylic acid cycle), thus affecting energy metabolism; on the other hand, branched chain amino acids form BC acyl CoA (branched chain acyl coenzyme A) under the catalysis of ketoacid dehydrogenase and participate in TCA cycle through succinyl‐coenzyme A, affecting the energy metabolism of the body (Biswas et al., 2019). In this experimental condition, the valine, leucine, and isoleucine, aminoacyl‐tRNA biosynthesis metabolism were affected by medium dose of XM, and the differential metabolite was leucine in which the content of leucine was decreased. It is speculated that more branched chain amino acids may be used as substrates to form ketones to participate in TCA cycle, so as to regulate tissue energy metabolism.

#### 4.4.4 Purine metabolism

Purine exists mostly in the form of nucleotide in the body. Purine nucleotides can be synthesized by de novo synthesis and salvage pathway, which requires a large amount of ATP. ATP is decomposed to produce ADP, which is hydrolyzed to AMP under the action of muscle kinase, and AMP generates hypoxanthine nucleotide (IMP) and NH_3_ under the action of adenylate deaminase (Liu & Mao, 1999). On the one hand, IMP can synthesize AMP again after obtaining amino group, which is also a part of purine nucleotide cycle. On the other hand, IMP can decompose into xanthine under the action of hypoxanthine nucleotide dehydrogenase and finally produce uric acid to be excreted out of the body (Li et al., 2018). In this experimental condition, both medium dose and low dose of XM could affect purine metabolism pathway, and their differential metabolites were xanthosine and AMP, respectively. Xanthosine is derived from the oxidative decomposition of xanthine nucleotides. AMP is not only a synthesis product of purine nucleotides, but also a product of its oxidative decomposition. In addition, AMP can obtain high‐energy phosphoric acid groups through oxidative phosphorylation to generate ATP. In this experiment, the content of xanthosine nucleoside decreased, and the content of AMP increased. It is speculated that after XM intervention, it can promote the circulation of purine nucleotides, reduce the oxidation decomposition of IMP into xanthine, and increase IMP to resynthesize AMP.

#### 4.4.5 Pentose phosphate metabolism

It is reported (Yao et al., 2020) that sugar can synthesize energy through the TCA cycle under aerobic respiration conditions and can form lactic acid through anaerobic glycolysis under anaerobic respiration conditions to provide energy for the body. In the conditions of this experiment, the high dose XM was involved in pentose phosphate metabolism. The different metabolite was gluconic acid, in which the content was decreased. The carbohydrates are converted into pyruvate through anaerobic or aerobic oxidation and participate in the TCA cycle and oxidative phosphorylation to provide energy for the body and accelerate the body's energy metabolism.

### 4.5 Effect of XM on the expression of Ampk, Sirt1, Pgc‐1α, and Ppar‐α mRNA

AMPK is an energy sensor in eukaryotic cells and plays an important role in maintaining the body's energy balance. Studies have confirmed that AMPK activation can increase mitochondrial biosynthesis (Ha et al., 2016; Herzig & Shaw, 2018; Lee & Kim, 2018; Shaw et al., 2004). PGC‐1α can not only increase energy consumption, but also increase the biogenesis and respiration rate of mitochondria, absorb, and use substrates to produce energy (Lehman et al., 2000; Stpierre et al., 2003; Wu et al., 1999). SIRT1 is a nicotinamide adenine dinucleotide (NAD^+^)‐dependent deacetylase, which can sense the energy metabolism state of living cells in tissues and affect the energy metabolism of tissues (Zhang et al., 2009). SIRT1, AMPK, and PGC‐1α can interact to form a network that can sense energy changes (Wen et al., 2016). AMPK can increase the activity of SIRT1 by increasing the level of NAD^+^, activate the deacetylation of SIRT1 downstream proteins (such as PGC‐1α), and increase the activity of PGC‐1α transcription, thereby regulating energy metabolism and mitochondrial synthesis (Chau et al., 2010; Scarpulla, 2011; Tang, 2016). AMPK can regulate the expression of downstream target molecule PPAR‐α, forming AMPK/PPAR‐α signaling pathway, which plays a very important role in maintaining energy metabolism (Wang et al., 2019). The results of this experiment showed that XM can increase the expression of SIRT1, AMPK, PGC‐1α, and PPAR‐α mRNA, indicating that XM may affect the body's energy metabolism in the following three ways: (a) XM may increase NAD^+^ level by activating AMPK and then activate SIRT1. SIRT1 can catalyze the acetylation of PGC‐1α, promote the biosynthesis of mitochondria, thereby affecting energy metabolism; (b) XM may directly activate PGC‐1α through AMPK, promote the synthesis of mitochondria, and increase the body's energy metabolism; (c) XM may promote energy metabolism through AMPK/PPAR‐α signaling and then affect glycolipid metabolism.

## 5 CONCLUSION

In conclusion, XM can enhance the kidney tissue energy metabolism. It may increase energy metabolism by changing the rate of mitochondrial metabolism and the number of mitochondria. And these effects may be mediated by TCA cycle metabolism, butanoate, propanoate metabolism, alanine, aspartate, and glutamate metabolism, retinol metabolism, purine metabolism, pentose phosphate metabolism, aminoacyl‐tRNA biosynthesis, valine, leucine, and isoleucine metabolism pathways, as well as by increasing expression of upstream genes Ampk, Sirt1, Ppar‐α, and Pgc‐1α mRNA.

## CONFLICTS OF INTEREST

The authors declared no potential conflicts of interest with respect to the research, authorship, and publication of this article.

## ETHICAL APPROVAL

The study was approved by the Experimental Animal Ethics Sub‐Committee of the Academic Committee of Jiangxi University of Traditional Chinese Medicine and complies with the animal research guidelines of the China Ethics Committee (JZLLSC2019‐0082).

## Data Availability

All datasets presented in this study are included in the article.

## References

[fsn32573-bib-0001] Acevedo, R. M. , Maiale, S. J. , Pessino, S. C. , Bottini, R. , Ruiz, O. A. , & Sansberro, P. A. (2013). A succinate dehydrogenase flavoprotein subunit‐like transcript is upregulated in *Ilex paraguariensis* leaves in response to water deficit and abscisic acid. Plant Physiology and Biochemistry, 65(Complete), 48–54. 10.1016/j.plaphy.2012.12.016 23416495

[fsn32573-bib-0002] Acin‐Perez, R. , Hoyos, B. , Zhao, F. , Vinogradov, V. , Fischman, D. A. , Harris, R. A. , Leitges, M. , Wongsiriroj, N. , Blaner, W. S. , Manfredi, G. , & Hammerling, U. (2010). Control of oxidative phosphorylation by vitamin A illuminates a fundamental role in mitochondrial energy homoeostasis. The FASEB Journal, 24(2), 627–636. 10.1096/fj.09-142281 19812372 PMC2812036

[fsn32573-bib-0003] Bao, H. Z. , Zhao, J. N. , Song, J. , & Li, Z. L. (2011). Research on long term toxicity of ethanol extracts of *Curculigo orchioides* Gaertn. Pharmacology and Clinics of Chinese Materia Medica, 3, 70–73.

[fsn32573-bib-0004] Bao, W. M. , Guo, Y. Z. , Tang, Y. M. , Lu, X. Q. , Sun, H. , & Zhao, X. W. (2004). The effect of cold/warm ischemia reperfusion injury on cell death pattern and the activities of Na^+^‐K^+^‐ATPase, Mg^2+^‐ATPase, Ca^2+^‐ATPase in liver of rats. Chinese Journal of Current Advances in General Surgery, 7(6), 343–346. 10.3969/j.issn.1009-9905.2004.06.009

[fsn32573-bib-0005] Biswas, D. , Duffley, L. , & Pulinilkunnil, T. (2019). Role of branched‐chain amino acid‐catabolizing enzymes in intertissue signaling, metabolic remodeling, and energy homeostasis. The FASEB Journal, 33(8), 8711–8731. 10.1096/fj.201802842RR 31084571

[fsn32573-bib-0006] Chau, M. D. L. , Gao, J. , Yang, Q. , Wu, Z. , & Keating, G. M. T. (2010). Fibroblast growth the factor 21 regulates energy metabolism by activating the AMPK‐SIRT1‐PGC‐1α pathway. Proceedings of the National Academy of Sciences of the United States of America, 107(28), 12553–12558. 10.2307/20724292 20616029 PMC2906565

[fsn32573-bib-0007] Chauhan, N. S. , Sharma, V. , Thakur, M. , & Dixit, V. K. (2010). Curculigo orchioides: The black gold with numerous health benefits. Zhong Xi Yi Jie He Xue Bao, 8(7), 613. 10.3736/jcim20100703 20619136

[fsn32573-bib-0008] Chen, H. L. (2011). Experimental study on toxicity of extract from curculigo orchioides gaertn. Qufu Normal University.

[fsn32573-bib-0009] Chen, L. M. , Jiang, E. , Guan, Y. M. , Xu, P. , Shen, Q. , Liu, Z. Y. , Zhu, W. F. , Chen, L. H. , Liu, H. N. , & Dong, H. H. (2020). Safety of high‐dose Puerariae Lobatae Radix in adolescent rats based on metabolomics. Food Science & Nutrition, 9(10), 1–17. 10.1002/fsn3.2044 PMC7866568 33598164

[fsn32573-bib-0010] Ekmekcioglu, C. , Strauss‐Blasche, G. , J. Leibetseder, V. , & Marktl, W. (1999). Toxicological and biochemical effects of different beverages on human intestinal cells. Food Research International, 32(6), 421–427. 10.1016/S0963-9969(99)00101-5

[fsn32573-bib-0011] Fan, Z. Y. (2010). Research on energy metabolism in rats which is affected by aconite, curculigo, coptis, phellodendron. Shandong University of Traditional Chinese Medicine.

[fsn32573-bib-0012] Ha, B. G. , Moon, D.‐S. , Kim, H. J. , & Shon, Y. H. (2016). Magnesium and calcium‐enriched deep‐sea water promotes mitochondrial biogenesis by Ampk‐activated signals pathway in 3T3‐L1 preadipocytes. Biomedicine & Pharmacotherapy, 83, 477–484. 10.1016/j.biopha.2016.07.009 27434863

[fsn32573-bib-0013] Hammerling, U. (2016). Retinol as electron carrier in redox signaling, a new frontier in vitamin A research. Hepatobiliary Surgery & Nutrition, 5(1), 15. 10.3978/j.issn.2304-3881.2016.01.02 26904553 PMC4739943

[fsn32573-bib-0014] Herzig, S. , & Shaw, R. J. (2018). AMPK: Guardian of metabolism and mitochondrial homeostasis. Nature Reviews Molecular Cell Biology, 19(2), 121–135. 10.1038/nrm.2017.95 28974774 PMC5780224

[fsn32573-bib-0015] Higashi, T. , Hayashi, H. , Kaida, T. , Arima, K. , Takeyama, H. , Taki, K. , Izumi, D. , Tokunaga, R. , Kosumi, K. , Nakagawa, S. , Okabe, H. , Imai, K. , Nitta, H. , Hashimoto, D. , Chikamoto, A. , Beppu, T. , & Baba, H. (2015). Prognostic impact of visceral fat amount and branched‐chain amino acids (BCAA) in hepatocellular carcinoma. Annals of Surgical Oncology, 22(3), 1041–1047. 10.1245/s10434-015-4796-5 26305023

[fsn32573-bib-0016] Hu, J. X. , Jiayu Gao, J. Y. , Zhao, Z. J. , & Yang, X. (2020). Response surface optimization of polysaccharide extraction from Galla Chinensis and determination of its antioxidant activity in vitro. Ciência E Tecnologia De Alimentos, 41(1), 188–194. 10.1590/fst.38619

[fsn32573-bib-0017] Kan, D. F. (2018). The effect and mechanisms of astragalus and its components on energy metabolism of rat hepatocytes. Shandong University of Traditional Chinese Medicine.

[fsn32573-bib-0018] Kim, Y. K. , & Hammerling, U. (2020). The mitochondrial pkcδ/retinol signal complex exerts real‐time control on energy homeostasis. BBA ‐ Molecular and Cell Biology of Lipids, 1865(11), 158614. 10.1016/j.bbalip.2020.158614 31927141 PMC7347429

[fsn32573-bib-0019] Lee, M. S. , & Kim, Y. (2018). Effects of isorhamnetin on adipocyte mitochondrial biogenesis and AMPK activation. Molecules, 23, 1853. 10.3390/molecules23081853 PMC6222361 30044453

[fsn32573-bib-0020] Lehman, J. J. , Barger, P. M. , Kovacs, A. , Saffitz, J. E. , Medeiros, D. M. , & Kelly, D. P. (2000). Peroxisome proliferator‐activated receptor gamma coactivator‐1 promotes cardiac mitochondrial biogenesis. Journal of Clinical Investigation, 106, 847–856. 10.1172/JCI10268 PMC517815 11018072

[fsn32573-bib-0021] Li, D. D. (2018). Changes in levels of xanthine oxidase and inflammatory factors in gout and hyperuricemia. China's Naturopathy, 26(12), 92–93. 10.19621/j.cnki.11-3555/r.2018.1259

[fsn32573-bib-0022] Li, M. , Cheng, L. F. , Aping Liu, A. P. , Tian, M. , & Wang, B. (2018). Effects of warm‐heating Chinese herbs on cold and heat sensitive proteins TRPM8 and TRPV1 in dorsal root ganglion. Pharmacology and Clinics of Chinese Materia Medica, 34(02), 55–58. 10.13412/j.cnki.zyyl.2018.02.015

[fsn32573-bib-0023] Li, M. , Zhang, B. , & Liu, X. Q. (2012). Effect of Curculiginis Rhizoma on metabolism and endocrine with “yang” deficiency syndrome in rats. Chinese Traditional Patent Medicine, 34(6), 1011–1013.

[fsn32573-bib-0024] Lingrel, J. B. (2010). The physiological significance of the cardiotonic steroid/ouabain‐binding site of the Na^+^‐K^+^‐ATPase. Annual Review of Physiology, 72(1), 395–412. 10.1146/annurev-physiol-021909-135725 PMC3079441 20148682

[fsn32573-bib-0025] Liu, J. Z. , Gao, W. X. , Luo, D. C. , & Cai, C. M. (1988). Characteristic of energy synthesis in brain mitochondria of rats exposed to hypoxia. Journal of Third Military Medical University, 06, 540.

[fsn32573-bib-0026] Liu, W. , & Mao, L. J. (1999). Effects of Intensive Exercise on purine nucleotide metabolism in skeletal muscle. Shandong Sports Science and Technology, 2, 36–38.

[fsn32573-bib-0027] Ma, R. S. , Zhang, R. Z. , Huang, H. Y. , Tan, Q. Y. , Yue, M. , & Zhao, S. H. (2019). Effects of dimethyl phthalate exposure on Ca^2+^‐Mg^2+^‐ATPase and Na^+^‐K^+^‐ATPase activity in liver of mice. Journal of Environment and Health, 36(07), 583–585. 10.16241/j.cnki.1001-5914.2019.07.005

[fsn32573-bib-0028] Neinast, M. D. , Jang, C. , Hui, S. , Murashige, D. S. , Chu, Q. , Morscher, R. J. , Li, X. , Zhan, L. E. , White, E. , Anthony, T. G. , Rabinowitz, J. D. , & Arany, Z. (2019). Quantitative analysis of the whole‐body metabolic fate of branched‐chain amino acids. Cell Metabolism, 29(2), 417–429. 10.1016/j.cmet.2018.10.013 30449684 PMC6365191

[fsn32573-bib-0029] Patel, M. S. , & Korotchinka, L. G. (2006). Regulation of the pyruvate dehydrogenase complex. Biochemical Society Transactions, 34, 217–222. 10.1042/BST20060217 16545080

[fsn32573-bib-0030] Patel, M. S. , & Roche, T. E. (1990). Molecular biology and biochemistry of pyruvate dehydrogenase complexes. FASEB Journal, 4, 3224–3233. 10.1096/fasebj.4.14.2227213 2227213

[fsn32573-bib-0031] Peng, S. L. , Liu, X. W. , Zhang, Z. R. , Yang, J. , He, H. Y. , & Xiao, W. P. (2010). Investigation of therapeutic mechanism of Weiweifang on experimental gastric ulcer in rats viewing from metabonomics. Chinese Journal of Integrated Traditional and Western Medicine, 30(10), 1073. 21066893

[fsn32573-bib-0032] Scarpulla, R. C. (2011). Metabolic control of mitochondrial biogenesis through the PGC‐1α family regulatory network. Biochimica Et Biophysica Acta (BBA)/Molecular Cell Research, 1813(7), 1269–1278. 10.1016/j.bbamcr.2010.09.019 PMC3035754 20933024

[fsn32573-bib-0033] Shaw, R. J. , Kosmatka, M. , Bardeesy, N. , Hurley, R. L. , Witters, L. A. , DePinho, R. A. , & Cantley, L. C. (2004). The tumor suppressor Lkb1 kinase directly activates AMP‐activated kinase and regulates apoptosis in response to energy stress. Proceedings of the National Academy of Sciences of the United States of America, 101(10), 3329–3335. 10.1073/pnas.0308061100 14985505 PMC373461

[fsn32573-bib-0034] Stpierre, J. , Lin, J. , Krauss, S. , Tarr, P. T. , Yang, R. , Newgard, C. B. , & Spiegelman, B. M. (2003). Bioenergetic analysis of peroxisome proliferator‐activated receptor gamma coactivators 1alpha and 1beta (PGC‐1alpha and PGC‐1beta) in muscle cells. Journal of Biological Chemistry, 278, 26597–26603. 10.1074/jbc.M301850200 12734177

[fsn32573-bib-0035] Sudar, E. , Velebit, J. , Gluvic, Z. , Zakula, Z. , Lazic, E. , Vuksanovic‐Topic, L. , Putnikovic, B. , Neskovic, A. , & Isenovic, E. R. (2008). Hypothetical mechanism of sodium pump regulation by estradiol under primary hypertension. Journal of Theoretical Biology, 251(4), 584–592. 10.1016/j.jtbi.2007.12.023 18304583

[fsn32573-bib-0036] Tang, B. L. (2016). Sirt1 and the mitochondria. Molecules and Cells, 39(2), 87–95. 10.14348/molcells.2016.2318 26831453 PMC4757807

[fsn32573-bib-0037] Wang, J. B. , Jin, C. , Xiao, X. H. , & Zhao, Y. L. (2008). Review and thinking of study of nature of Chinese material medica. China Journal of Traditional Chinese Medicine and Pharmacy, 23(7), 572–576.

[fsn32573-bib-0038] Wang, N. , Zhang, N. , Li, T. , Wang, M. , Huang, X. , & Liu, S. Y. (2020). Untargeted metabolomics study of ginseng in treatment of spleen‐Qi deficiency. China Journal of Chinese Materia Medica, 45(2), 398–404. 10.19540/j.cnki.cjcmm.20191017.201 32237324

[fsn32573-bib-0039] Wang, Q. , Chen, X. , Xie, Z. X. , & Liu, X. F. (2020). Untargeted metabolomics of genetically modified cows expressing lactoferrin based on serum and milk. Journal of Agricultural and Food Chemistry, 68(2), 686–696. 10.1021/acs.jafc.9b06630 31877248

[fsn32573-bib-0040] Wang, X. Y. , Gong, X. Y. , Zhang, H. N. , Zhu, W. S. , Jiang, Z. W. , Yujing Shi, Y. J. , & Li, L. (2020). In vitro anti‐aging activities of ginkgo biloba leaf extract and its chemical constituents. Ciência E Tecnologia De Alimentos, 40(2), 476–482. 10.1590/fst.02219

[fsn32573-bib-0041] Wang, X. , Sun, H. , Zhang, A. , Sun, W. , Ping, W. , & Wang, Z. (2011). Potential role of metabolomics approaches in the area of traditional Chinese medicine: As pillars of the bridge between Chinese and western medicine. Journal of Pharmaceutical & Biomedical Analysis, 55(5), 859–868. 10.1016/j.jpba.2011.01.042 21353755

[fsn32573-bib-0042] Wang, Z. H. , Jia, C. S. , Wang, W. H. , Tong, W. , & Jiang, Y. B. (2020). Effects of low temperature storage on energy metabolism, related physiology and quality in ‘Jinhong’ apple fruit. Acta Horticulturae Sinica, 47(12), 2277–2289. 10.16420/j.issn.0513-353x.2020-0418

[fsn32573-bib-0043] Wang, Z. , Li, J. B. , Dong, X. , & Shen, X. X. (2019). Effect of Buyang Huanwu Tang on myocardial mitochondrial energy metabolism and AMPK/PPARα signaling pathway in rats with diastolic heart failure. Chinese Journal of Experimental Traditional Medical Formulae, 9, 12–17. 10.13422/j.cnki.syfjx.20190902

[fsn32573-bib-0044] Wen, C. Y. , Duan, Y. H. , Li, Y. H. , Guo, Q. P. , Kong, X. F. , & Li, F. N. (2016). Energy sensing network ampk/sirt1/pgc‐lα involved in regulating skeletal muscle fiber type transformation. Chinese Journal of Animal Nutrition, 28(1), 57–63. 10.3969/j.issn.1006-267x.2016.01.009

[fsn32573-bib-0045] Wu, L. J. , Yu, X. C. , Ke, J. Q. , Deng, S. Y. , Hu, C. , Xiong, Y. H. , & Tang, X. L. (2021). Research progress in the treatment of chemical liver injure with traditional Chinese medicine based on metabolomics. Chinese Journal of Experimental Traditional Medical Formulae, 27(12), 202–215. 10.13422/j.cnki.syfjx.20210716

[fsn32573-bib-0046] Wu, Z. , Puigserver, P. , Andersson, U. , Zhang, C. , Adelmant, G. , Mootha, V. , Troy, A. , Cinti, S. , Lowell, B. , Scarpulla, R. C. , & Spiegelman, B. M. (1999). Mechanisms controlling mitochondrial biogenesis and respiration through the thermogenic coactivator PGC‐1α. Cell, 98, 115–124. 10.1016/S0092-8674(00)80611-X 10412986

[fsn32573-bib-0047] Xiang, L. H. , Chen, Y. P. , Zhang, Z. , Shan, Z. Y. , Yu, Z. M. , & Lv, A. P. (2006). Effect of long‐term toxicity test of 24 poisonous Chinese herbs on viscera index in rats. Journal of Basic Chinese Medicine, 1(6), 35–36.

[fsn32573-bib-0048] Xu, J. , Cheng, K. K. , Yang, Z. , Wang, C. , Shen, G. , Wang, Y. , Liu, Q. , & Dong, J. (2015). ^1^H‐NMR metabolic profiling of biofluids from rats with gastric mucosal lesion and electroacupuncture treatment. Evidence Based Complementary & Alternative Medicine, 34, 801691. 10.1155/2015/801691 PMC4485499 26170882

[fsn32573-bib-0049] Yang, H. (2012). Research on chemical constituents and activity of curculigo orchioides. Hunan University of Traditional Chinese Medicine.

[fsn32573-bib-0050] Yao, M. X. , Ran, S. , Sun, F. F. , Song, Y. , Wang, X. L. , & Han, Y. Q. (2020). Mechanism of processing‐compatibility of ginger in treating cold asthma rat by gas chromatography‐mass spectrometry based on metabolomics. Chinese Traditional and Herbal Drugs, 51(3), 118–130. 10.7501/j.issn.0253-2670.2020.03.017

[fsn32573-bib-0051] Zhai, M. L. , Yu, M. , & Wang, C. X. (2016). Pathological mechanism of spleen yang deficiency syndrome based on energy metabolism related enzyme activity. Journal of Liaoning University of Traditional Chinese Medicine, 18(09), 90–92. 10.13194/j.issn.1673-842x.2016.09.026

[fsn32573-bib-0052] Zhang, H. S. , Zhou, Y. , & Xu, F. (2009). AMPK, SIRT1, and energy metabolism. Journal of Clinical and Pathological Research, 3, 24–28. 10.3969/j.issn.1673-2588.2009.03.004

[fsn32573-bib-0053] Zhang, Z. , Shan, Z. Y. , Xiang, L. H. , Chen, Y. P. , Yu, Z. M. , & Lv, A. P. (2005). Effects of long‐term administration of 24 poisonous Chinese herbs on blood biochemical indexes in rats. Journal of Basic Chinese Medicine, 11(12), 918–919.

[fsn32573-bib-0054] Zhao, Y. B. , Guo, Y. X. , Chen, Y. Q. , Liu, S. , Wu, N. , & Jia, D. L. (2020). Curculigoside attenuates myocardial ischemia‐reperfusion injury by inhibiting the opening of the mitochondrial permeability transition pore. International Journal of Molecular Medicine, 45(5), 1514–1524. 10.3892/ijmm.2020.4513 32323742 PMC7138276

[fsn32573-bib-0055] Zheng, Q. F. (2015). The objectivity study on the “hot” property of aconiti lateralis radix based on the energy metabolism of mitochondrion. Chengdu University of Traditional Chinese Medicine.

[fsn32573-bib-0056] Zhou, X. Y. , Wang, G. Y. , Lai, L. , Xu, L. , Shen, Q. P. , Wang, Y. J. , Fan, M. , & Shen, L. (2021). Traditional Chinese medicine diet paratherapy for alleviating toxicity in chemotherapy and radiotherapy in cancer patients: A meta‐analysis. Ciência E Tecnologia De Alimentos, (6), 1–10. 10.1590/fst.31720

[fsn32573-bib-0057] Zhou, Y. Z. , Xu, G. , Ju, C. G. , & Jia, T. Z. (2014). Study on “heat by heat gain” effect of wine‐broiled Curculigo orchioides. Chinese Traditional and Herbal Drugs, 45(10), 1434–1438. 10.7501/j.issn.0253-2670.2014.10.015

